# Mineralogy, morphology, and reaction kinetics of ureolytic bio-cementation in the presence of seawater ions and varying soil materials

**DOI:** 10.1038/s41598-022-21268-3

**Published:** 2022-10-12

**Authors:** Robert J. Burdalski, Bruna G. O. Ribeiro, Michael G. Gomez, Drew Gorman-Lewis

**Affiliations:** 1 Department of Civil and Environmental Engineering, University of Washington, Seattle, WA 98195 USA; 2 Department of Earth and Space Sciences, University of Washington, Seattle, WA 98195 USA

**Keywords:** Civil engineering, Applied microbiology, Biogeochemistry, Natural hazards

## Abstract

Microbially-induced calcium carbonate precipitation (MICP) is a bio-cementation process that can improve the engineering properties of granular soils through the precipitation of calcium carbonate (CaCO_3_) minerals on soil particle surfaces and contacts. The technology has advanced rapidly as an environmentally conscious soil improvement method, however, our understanding of the effect of changes in field-representative environmental conditions on the physical and chemical properties of resulting precipitates has remained limited. An improved understanding of the effect of subsurface geochemical and soil conditions on process reaction kinetics and the morphology and mineralogy of bio-cementation may be critical towards enabling successful field-scale deployment of the technology and improving our understanding of the long-term chemical permanence of bio-cemented soils in different environments. In this study, thirty-five batch experiments were performed to specifically investigate the influence of seawater ions and varying soil materials on the mineralogy, morphology, and reaction kinetics of ureolytic bio-cementation. During experiments, differences in reaction kinetics were quantified to identify conditions inhibiting CaCO_3_ precipitation and ureolysis. Following experiments, scanning electron microscopy, x-ray diffraction, and chemical composition analyses were employed to quantify differences in mineralogical compositions and material morphology. Ions present in seawater and variations in soil materials were shown to significantly influence ureolytic activity and precipitate mineralogy and morphology, however, calcite remained the predominant CaCO_3_ polymorph in all experiments with relative percentages exceeding 80% by mass in all precipitates.

## Introduction

Commercially available ground improvement methods oftentimes rely on high mechanical energy and/or energy-intensive materials, such as portland cement, to improve the engineering properties of soils^1–5^. Recently the emerging field of bio-mediated soil improvement has demonstrated the potential of biogeochemical processes to enable comparable engineering improvements with significant reductions in detrimental environmental impacts^6–9^. Microbially-induced calcium carbonate precipitation (MICP) is one such technology, which involves the precipitation of calcium carbonate (CaCO_3_) minerals on soil particle surfaces and contacts following the biologically mediated hydrolysis of urea in the presence of soluble calcium (Ca^2+^)^10–12^. MICP has the ability to transform the engineering properties of soils by increasing initial shear stiffness, strength, and thermal conductivity, while reducing soil hydraulic conductivity and porosity^13–20^. The bio-mediated process can address a diverse range of engineering applications including liquefaction mitigation, slope stability improvement, subsurface flow manipulation, construction material development, and contaminant immobilization^8,12,21–24^. Despite many recent advances in the technology including improved characterization of engineering behaviors^14,25–28^ and successful demonstration at meter-scale^29–36^, our collective understanding of the impact of environmental conditions on the bio-cementation process and the physical and chemical properties of bio-cemented soils including mineralogy, crystal morphology, chemical composition, and solubility has remained limited.

Although bio-cementation is commonly referred to as consisting of exclusively calcite, CaCO_3_ can exist as different mineral polymorphs each with varying atomic structures, morphologies, and physicochemical properties^37^. The mineralogy of generated CaCO_3_ will be a critical factor governing the long-term chemical permanence of bio-cementation once employed at field-scale as CaCO_3_ mineral polymorphs have solubilities that can span orders of magnitude. For example, at 1 atm and 25 °C, calcite has the lowest polymorph solubility product (K_sp_) of 10^−8.48^, however, aragonite (10^−8.34^), vaterite (10^−7.91^), ikaite (10^−6.62^), and amorphous calcite (10^−6.40^) each have solubilities that are considerably higher, despite all minerals being CaCO_3_^38–40^. Thus, an improved understanding of the influence of chemical conditions during MICP, including those imposed by treatment solution compositions and environmental conditions, on the mineralogy and morphology of generated bio-cementation may have important implications towards evaluating and optimizing the long-term permanence and engineering performance of bio-cemented soils. Past researchers have considered an extensive range of different chemical reagents and concentrations in applied bio-cementation treatment solutions, however, almost all treatment solutions have included supplied urea (for microbial CO_3_^2−^ production), soluble Ca^2+^ (for CaCO_3_ precipitation), and microbial nutrients and substrates (to maintain and/or increase ureolytic cell densities). Occasionally, other reactants have also been supplied to enable control of reaction rates (e.g., NH_4_Cl), microbial enrichment (e.g., NaOH, NH_4_Cl, sodium acetate), and/or alter other environmental conditions (e.g., NaHCO_3_)^29,41–47^. While continued optimization of treatment solution compositions was to be expected as the technology has matured, large variations in applied treatment solutions continue to persist even between the most recent studies suggesting that researchers have yet to reach firm conclusions regarding the effect of treatment solution compositions and environmental conditions on the bio-cementation process^8,20^.

Although changes in treatment solution compositions afford the opportunity to directly influence chemical conditions during CaCO_3_ precipitation, environmental conditions including subsurface groundwater chemistry and in situ soil mineralogy present unavoidable site-specific factors governed by application location. An improved understanding of how these conditions may alter reaction kinetics and generated mineral products will have important practical implications, guiding employed treatment solution compositions, allowing for the identification of favorable application locations, and enabling improved assessment of long-term material permanence. A multitude of environmental conditions may be present at any given site including differences in available cations, anions, solution pH, microorganisms, and other factors, however, a clear opportunity exists to apply the MICP process to marine and brackish environments wherein elevated total alkalinity and calcium concentrations may permit improved permanence^48^. Past studies have examined the effect of seawater on MICP-treated soils with most focusing on investigating differences in achieved mechanical properties. Mortenson et al.^44^ found that higher shear wave velocities could be achieved when MICP was performed in seawater due to its higher total alkalinity and calcium concentrations. Cheng et al.^49^ found that higher unconfined compressive strengths could be obtained for the same CaCO_3_ content when seawater was used to prepare treatment solutions, however, permeability reductions remained similar to samples treated with freshwater at comparable cementation levels. Similarly, Yu and Rong^50^ observed increases in the unconfined compressive strength of cemented sand blocks when seawater-based solutions were employed. In contrast, Miftah et al.^51^ found that seawater had minimal effects on the strength, CaCO_3_ content, and mineralogy of precipitates obtained using enzyme-induced calcium carbonate precipitation (EICP) in a beach sand material. Although the majority of these studies suggest that the presence of seawater may have beneficial effects on the mechanical improvements afforded by MICP, such studies have provided limited insights regarding which specific ions in seawater may influence microbial activity and precipitation kinetics and how the mineralogy and composition of generated precipitates may shift as a function these differences.

Recent investigations have also expanded the range of soil materials considered suitable for the MICP process beyond the poorly-graded sand materials for which the majority of past studies have been performed. These investigations have involved various natural sand mixtures^36,52–54^, clay minerals^55,56^, peaty soils^57,58^, and mine wastes^59–61^, however, almost all of these studies have involved differences in treatment solutions, preparation methods, and application and testing procedures, thereby rendering the effect of changes in soil mineralogy difficult to isolate from other experimental variables. Most these studies have also solely investigated changes in macroscale engineering behaviors (i.e., peak shear strength, shear wave velocity) which would not be expected to resolve important differences in precipitate microstructure and composition nor provide insights regarding changes in reaction behaviors. In order to more effectively understand the effect of specific environmental factors on the bio-cementation process, systematic experimentation must be performed which minimizes differences in biological, chemical, and physical factors.

Although not yet fully explored for bio-cementation, abiotic CaCO_3_ synthesis experiments completed under more controlled conditions suggest that even small changes in solution chemistry can dramatically alter precipitate formation including the presence of various ions found in seawater (e.g., SO_4_^2−^, Na^+^, Mg^2+^) and associated with common soil minerals. For example, Berner^62^, Zhang and Dawe^63^, and Meldrum and Hyde^64^ observed large changes in CaCO_3_ precipitation kinetics and precipitate morphology when trace concentrations of magnesium (Mg^2+^) were present. Busenberg and Plummer^65^ found that sulfate (SO_4_^2−^) concentrations in seawater (≈ 28 mM) significantly inhibited CaCO_3_ precipitation rates and that both SO_4_^2−^ and Na^+^ were incorporated within CaCO_3_ when precipitation occurred in artificial seawater. The presence of manganese (Mn^2+^), strontium (Sr^2+^), and phosphate (PO_4_^3−^) have also been identified as important factors influencing CaCO_3_ precipitation^66^. Furthermore, differences in mineral surface chemistry have also long been recognized by researchers as having a significant impact on carbonate mineral precipitation including influencing the particular mineral polymorphs formed as well as crystal nucleation and rates of formation^67^. For example, Liu et al.^68^ found that the presence of clay minerals can influence abiotic CaCO_3_ and dolomite (CaMg(CO_3_)_2_) formation, with illite and montmorillonite clay minerals accelerating carbonate mineral formation, but kaolinite exhibiting more minimal effects on such reactions. Although such studies have yielded important insights regarding the effect of soil minerals and solution chemistry on abiotic mineral formation, the addition of biological catalysts during MICP may present further complications, thereby altering mineral formation in manners that are not thermodynamically predictable^69,70^. As the technology advances towards field-scale implementation, an improved understanding of the consequences of site-specific conditions will be increasingly important.

In this study, thirty-five small-scale batch experiments were conducted to investigate the effect of field-representative environmental conditions on the mineralogy, morphology, and reaction kinetics of ureolytic bio-cementation using augmented *Sporosarcina pasteurii* (*S. pasteurii*) bacteria. Experiments were completed in five different series and explored the effect of varying concentrations of artificial seawater, differences in Mg^2+^, Sr^2+^, SO_4_^2−^, and Na^+^ ion additions, and variations in soil materials on the MICP process and resulting CaCO_3_ precipitates. Results from these experiments provide new insights regarding the effect of soil materials and seawater ions on the reaction kinetics, morphology, and mineralogy of ureolytic bio-cementation, relevant towards improving our understanding of process deployment and material longevity when applied to different geochemical environments.

## Materials and methods

### Batch experiments

All experiments were conducted in 100 mm diameter, 15 mm deep, flat bottom glass petri dishes (Corning Inc.) which included 5.3 g of oven-dried soil and 45 mL of treatment solutions. The performed batch experiments provided several advantages relative to soil columns including: (1) permitting homogenous solution conditions to be achieved at the start of experiments, (2) eliminating the potential for changes in reaction kinetics and precipitation formation due to uncontrolled differences in cell attachment, reactions during injections, hydraulic conductivity differences, and other physical phenomena, and (3) minimizing the influence of solution sampling events on specimen saturation. In order to simulate field-representative treatment processes wherein soils are either first augmented or stimulated to achieve sufficient ureolytic activity and then subsequently bio-cemented^12,29,36,41,43,47^, dry soil masses were directly mixed with *S. pasteurii* cell suspensions (≈ 1 mL) prior to all experiments. After mixing, soil and cell suspensions were allowed to equilibrate at 1.6 °C for a minimum of 12 h prior to introducing treatment solutions in order to promote attachment of cells onto soil particle surfaces following other augmented studies^12,29,47,71^ while limiting microbial activity in the absence of supplied nutrients. Prepared treatment solutions (≈ 44 mL) were mixed with augmented soil mixtures after the equilibration period, vortexed at 800 rpm for 10 s, placed within plates, and then covered with parafilm to minimize solution-air interactions and the potential for evaporation. All experiments were allowed to react for up to 30 h at a constant temperature of 23 °C to allow ureolysis and precipitation reactions to achieve completion in most experiments. All experiments contained no supplied nutrients intended to mitigate the potential for cell growth and aerobic respiration during experiments as well as eliminate the presence of excess proteins and amino acids, which may have altered precipitate formation. Although efforts were made to minimize the potential for biological contamination, included soil materials were not strictly sterilized as all experiments lacked nutrients and were designed to minimize the potential for microbial growth. After all retention periods, remaining solutions were drained, collected, and frozen and bio-cemented soils were rinsed twice with absolute ethanol to remove soluble reaction byproducts and then oven-dried at 110 °C for 48 h. After drying, all bio-cemented soil specimens were stored in a vacuum desiccator at room temperature and a relative humidity of less than 10% and were stored for no more than 2 weeks before completing material analyses intended to mitigate the potential for subsequent mineralogical changes. Similar mineral preservation processes have been used successfully in other past studies involving CaCO_3_ minerals^72,73^.

### Experimental series

Thirty-five batch experiments (E1–E35) were completed in five different experimental series to examine the effect of changes in seawater ions and differences in soil materials on the bio-cementation process and resulting precipitates. Table 1 summarizes all batch experiments including experiment name, experimental series, soil material, treatment solution composition, augmented cell densities measured via OD_600_ measurements (direct measurements of cell densities), augmented cell densities estimated from observed urea hydrolysis activity and a PHREEQC kinetic model (urea hydrolysis activity based estimates of cell densities), the ratio between PHREEQC-estimated (activity based estimates) and OD_600_-measured cell densities (direct measurements), and the ratio between PHREEQC-estimated cell densities for experiments (activity-based estimates for experiments) and their respective controls for each series (activity-based estimates for respective control specimens). Experiments were augmented with cells at the same cell density using the same batch of growth media for each respective series, in order to minimize biological differences between experiments. For all experimental series, similar control experiments (E1, E4, E13, E16, E21, E25) were also repeated which contained only urea, Ca^2+^, augmented *S. pasteurii* cells, and Ottawa F-65 sand, in order to allow for comparison of reaction kinetics and precipitates between experiments while controlling for unavoidable variations in augmented cell activities. Experimental series 1 (E1–E3) examined bio-cementation in artificial seawater mixtures prepared at three different concentrations by volume. Experimental series 2 (E4–E11) examined bio-cementation in the presence of discrete Mg^2+^, Sr^2+^, and SO_4_^2−^ ion additions in order to further investigate the effect of specific seawater ion concentrations on the MICP process at concentrations between 50 and 200% that present in seawater. Experimental series 3 (E12–E20) examined the effect of discrete seawater ion additions on urea hydrolysis alone in experiments which contained minimal supplied Ca^2+^ (0 or 10 mM) and no significant precipitation. Experimental series 4 (E21–E24) further examined bio-cementation in the presence of Na^+^ ion additions intended to assess the effect of ionic strength increases relevant to earlier seawater experiments. Lastly, experimental series 5 (E25–E35) explored bio-cementation in the presence of eleven different soils of varying mineralogy.

**Table 1:** Summary of all batch experiments, treatment solution compositions, and augmented cell measurements and estimations.

Batch experiments			Treatment solution composition									
Specimen name	Experimental series	Soil material	Urea (mM)	Ca^2+^ (mM)	Mg^2+^ (mM)	SO_4_^2−^ (mM)	Sr^2+^ (mM)	Na^+^ (mM)	Cl^−^ (mM)	K^+^ (mM)	B^3+^ (mM)	CO_3_^2−^ mM)
E1_0% ASW	(1) ASW	Ottawa F-65 sand	250	250	–	–	–	3	503	–	–	–
E2_50% ASW	(1) ASW	Ottawa F-65 sand	250	255	27	14	0.05	242	782	2. 5	0.015	1.5
E3_100% ASW	(1) ASW	Ottawa F-65 sand	250	260	54	27	0. 10	481	1051	5	0. 03	3
E4_0 mM Mg^2+^/0 mM Sr^2+^	(2) ASW ions	Ottawa F-65 sand	250	250	–	–	–	3	503	–	–	–
E5_27 mM Mg^2+^	(2) ASW ions	Ottawa F-65 sand	250	250	27	–	–	3	557	–	–	–
E6_54 mM Mg^2+^	(2) ASW ions	Ottawa F-65 sand	250	250	54	–	–	3	611	–	–	–
E7_108 mM Mg^2+^	(2) ASW ions	Ottawa F-65 sand	250	250	108	–	–	3	719	–	–	–
E8_0.055 mM Sr^2+^	(2) ASW ions	Ottawa F-65 sand	250	250	–	–	0. 055	3	504	–	–	–
E9_0.11 mM Sr^2+^	(2) ASW ions	Ottawa F-65 sand	250	250	–	–	0. 11	3	504	–	–	–
E10_0.22 mM Sr^2+^	(2) ASW ions	Ottawa F-65 sand	250	250	–	–	0. 22	3	504	–	–	–
E11_14 mM SO_4_^2−^	(2) ASW ions	Ottawa F-65 sand	250	250	–	14		31	504	–	–	–
E12_100% ASW	(3) ASW ions (ureolysis only)	Ottawa F-65 sand	250	10	54	27	0. 10	481	539	5	0. 03	3
E13_0 mM SO_4_^2−^	(3) ASW ions (ureolysis only)	Ottawa F-65 sand	250	–	–	–	–	3	3	–	–	–
E14_14 mM SO_4_^2−^	(3) ASW ions (ureolysis only)	Ottawa F-65 sand	250	–	–	14	–	31	3	–	–	–
E15_28 mM SO_4_^2−^	(3) ASW ions (ureolysis only)	Ottawa F-65 sand	250	–	–	28	–	59	3	–	–	–
E16_0 mM Mg^2+^	(3) ASW ions (ureolysis only)	Ottawa F-65 sand	250	–	–	–	–	3	3	–	–	–
E17_54 mM Mg^2+^	(3) ASW ions (ureolysis only)	Ottawa F-65 sand	250	–	54	–	–	3	111	–	–	–
E18_54 mM Mg^2+^ + 10 mM Ca^2+^	(3) ASW ions (ureolysis only)	Ottawa F-65 sand	250	10	54	–	–	3	131	–	–	–
E19_108 mM Mg^2+^	(3) ASW ions (ureolysis only)	Ottawa F-65 sand	250	–	108	–	–	3	219	–	–	–
E20_108 mM Mg^2+^ + 10 mM Ca^2+^	(3) ASW ions (ureolysis only)	Ottawa F-65 sand	250	10	108	–	–	3	239	–	–	–
E21_0 mM Na^+^	(4) Sodium variations	Ottawa F-65 sand	250	250	–	–	–	3	503	–	–	–
E22_10 mM Na^+^	(4) Sodium variations	Ottawa F-65 sand	250	250	–	–	–	13	513	–	–	–
E23_100 mM Na^+^	(4) Sodium variations	Ottawa F-65 sand	250	250	–	–	–	103	603	–	–	–
E24_1000 mM Na^+^	(4) Sodium variations	Ottawa F-65 sand	250	250	–	–	–	1003	1503	–	–	–
E25_Ottawa sand	(5) Soil variations	Ottawa F-65 sand	250	250	–	–	–	3	503	–	–	–
E26_Fraser river sand	(5) Soil variations	Fraser river sand	250	250	–	–	–	3	503	–	–	–
E27_Concrete sand	(5) Soil variations	Concrete sand	250	250	–	–	–	3	503	–	–	–
E28_Covelo sand	(5) Soil variations	Covelo sand	250	250	–	–	–	3	503	–	–	–
E29_Delta sand	(5) Soil variations	Delta sand	250	250	–	–	–	3	503	–	–	–
E30_Monterey sand	(5) Soil variations	Monterey sand	250	250	–	–	–	3	503	–	–	–
E31_Feldspar	(5) Soil variations	Feldspar	250	250	–	–	–	3	503	–	–	–
E32_Olivine	(5) Soil variations	Olivine	250	250	–	–	–	3	503	–	–	–
E33_Mica	(5) Soil variations	Mica	250	250	–	–	–	3	503	–	–	–
E34_Kaolinite	(5) Soil variations	Kaolinite	250	250	–	–	–	3	503	–	–	–
E35_Montmorillonite	(5) Soil variations	Montmorillonite	250	250	–	–	–	3	503	–	–	–

Batch experiments			Augmented cells									
Specimen name	Experimental series	Soil material	OD_600_-based direct measurement of cell density (cells/mL)	PHREEQC activity-based estimate of cell density (cells/mL)	Ratio of PHREEQC activity-based estimated cell density to OD_600_-based direct measure of cell density	Ratio of PHREEQC activity-based estimated cell density for experiment to PHREEQC activity-based estimated cell density of control						
E1_0% ASW	(1) ASW	Ottawa F-65 sand	7.6E+07	1.3E+08	171%	100% (control)						
E2_50% ASW	(1) ASW	Ottawa F-65 sand	7.6E+07	1.2E+08	158%	92%						
E3_100% ASW	(1) ASW	Ottawa F-65 sand	7.6E+07	6.0E+07	79%	46%						
E4_0 mM Mg^2+^/0 mM Sr^2+^	(2) ASW ions	Ottawa F-65 sand	7.1E+07	1.0E+08	140%	100% (control)						
E5_27 mM Mg^2+^	(2) ASW ions	Ottawa F-65 sand	7.1E+07	5.5E+07	77%	55%						
E6_54 mM Mg^2+^	(2) ASW ions	Ottawa F-65 sand	7.1E+07	5.0E+07	70%	50%						
E7_108 mM Mg^2+^	(2) ASW ions	Ottawa F-65 sand	7.1E+07	4.5E+07	63%	45%						
E8_0.055 mM Sr^2+^	(2) ASW ions	Ottawa F-65 sand	7.1E+07	9.0E+07	126%	90%						
E9_0.11 mM Sr^2+^	(2) ASW ions	Ottawa F-65 sand	7.1E+07	8.5E+07	119%	85%						
E10_0.22 mM Sr^2+^	(2) ASW ions	Ottawa F-65 sand	7.1E+07	8.0E+07	112%	80%						
E11_14 mM SO_4_^2−^	(2) ASW ions	Ottawa F-65 sand	7.1E+07	8.5E+07	119%	85%						
E12_100% ASW	(3) ASW ions (ureolysis only)	Ottawa F-65 sand	6.6E+07	2.0E+07	30%	44%						
E13_0 mM SO_4_^2−^	(3) ASW ions (ureolysis only)	Ottawa F-65 sand	6.6E+07	4.5E+07	68%	100% (control)						
E14_14 mM SO_4_^2−^	(3) ASW ions (ureolysis only)	Ottawa F-65 sand	6.6E+07	4.5E+07	68%	100%						
E15_28 mM SO_4_^2−^	(3) ASW ions (ureolysis only)	Ottawa F-65 sand	6.6E+07	4.5E+07	68%	100%						
E16_0 mM Mg^2+^	(3) ASW ions (ureolysis only)	Ottawa F-65 sand	6.6E+07	4.5E+07	68%	100% (control)						
E17_54 mM Mg^2+^	(3) ASW ions (ureolysis only)	Ottawa F-65 sand	6.6E+07	2.5E+07	38%	56%						
E18_54 mM Mg^2+^ + 10 mM Ca^2+^	(3) ASW ions (ureolysis only)	Ottawa F-65 sand	6.6E+07	2.8E+07	42%	61%						
E19_108 mM Mg^2+^	(3) ASW ions (ureolysis only)	Ottawa F-65 sand	6.6E+07	1.5E+07	23%	33%						
E20_108 mM Mg^2+^ + 10 mM Ca^2+^	(3) ASW ions (ureolysis only)	Ottawa F-65 sand	6.6E+07	1.5E+07	23%	33%						
E21_0 mM Na^+^	(4) Sodium variations	Ottawa F-65 sand	8.0E+07	1.1E+08	131%	100% (control)						
E22_10 mM Na^+^	(4) Sodium variations	Ottawa F-65 sand	8.0E+07	1.1E+08	137%	105%						
E23_100 mM Na^+^	(4) Sodium variations	Ottawa F-65 sand	8.0E+07	1.1E+08	131%	100%						
E24_1000 mM Na^+^	(4) Sodium variations	Ottawa F-65 sand	8.0E+07	5.5E+07	69%	52%						
E25_Ottawa sand	(5) Soil variations	Ottawa F-65 sand	8.2E+07	1.3E+08	159%	100% (control)						
E26_Fraser river sand	(5) Soil variations	Fraser river sand	8.2E+07	7.5E+06	9%	6%						
E27_Concrete sand	(5) Soil variations	Concrete sand	8.2E+07	8.0E+07	98%	62%						
E28_Covelo sand	(5) Soil variations	Covelo sand	8.2E+07	4.5E+07	55%	35%						
E29_Delta sand	(5) Soil variations	Delta sand	8.2E+07	7.3E+07	89%	56%						
E30_Monterey sand	(5) Soil variations	Monterey sand	8.2E+07	1.1E+08	135%	85%						
E31_Feldspar	(5) Soil variations	Feldspar	8.2E+07	1.1E+08	129%	81%						
E32_Olivine	(5) Soil variations	Olivine	8.2E+07	1.0E+08	126%	79%						
E33_Mica	(5) Soil variations	Mica	8.2E+07	1.7E+08	208%	131%						
E34_Kaolinite	(5) Soil variations	Kaolinite	8.2E+07	1.7E+08	202%	127%						
E35_Montmorillonite	(5) Soil variations	Montmorillonite	8.2E+07	1.1E+08	135%	85%						

### Soil materials

All batch experiments were performed using Ottawa F-65 Sand with the exception of the soil varied experiments (experimental series 5), which involved ten additional soils. Ottawa F-65 Sand was selected for the majority of the batch experiments performed in this study due to its chemically inert quartz mineralogy, near uniform grain size distribution, low fines content, and extensive characterizations in past bio-cementation and other geotechnical studies^25,27,74–76^. Ottawa F-65 Sand has a D_10_ of 0.13 mm, a D_30_ of 0.18 mm, a D_60_ of 0.23 mm, no fines^75^, and classifies as a poorly-graded sand (SP) following ASTM D2487-17^77^. In experimental series 5, other soil materials were present including other poorly-graded natural and processed sands (Fraser River Sand, Covelo Sand, Delta Sand, Concrete Sand, Monterey Sand), low and high plasticity phyllosilicate minerals (mica, kaolinite, montmorillonite), and other common soil minerals (feldspar, olivine). Table 2 presents the material sources, USCS classifications, average particle sizes (D_50_), fines contents, and mineralogical composition of all soils used in this study as determined by XRD analyses.

**Table 2:** Summary of all tested soil materials.

Soil material	Source	USCS	D_50_ (mm)	Fines content (%)	XRD Characterization	
					Primary minerals	Other detectable minerals (> 2%)
Ottawa sand	Commerical (US Silica Inc.)	SP	0.2	–	Quartz (~ 100%)	–
Fraser river sand	Field Sample (Delta, BC, Canada)	SM	0.2	15.6	Quartz (~ 85%)	Albite (~ 15%)
Concrete sand	Teichert Aggregates (Woodland, CA, USA)	SP	1.2	1.1	Quartz (~ 75%)	Albite (~ 25%)
Covelo sand	DenBeste Supply (Ukiah, CA, USA)	SP	1.2	1.6	Quartz (~ 87%)	Albite (~ 13%)
Delta sand	NorCal Aggregates (Petaluma, CA, USA)	SP	0.3	1.3	Quartz (~ 58%)	Albite (~ 42%)
Monterey sand	Cemex Inc. (Marina, CA, USA)	SP	1.4	–	Quartz (~ 47%)	Microcline (~ 42%), Albite (~ 11%)
Feldspar	Laguna Clay (City of Industry, CA, USA)	SP	4.0	–	Microcline (~ 78%)	Albite (~ 12%), Quartz (~ 10%)
Olivine	Laguna Clay (City of Industry, CA, USA)	SP	2.4	–	Forsterite (~ 90%)	Fayalite (~ 10%)
Mica	Laguna Clay (City of Industry, CA, USA)	SP	2.6	–	Lepidolite (~ 100%)	–
Kaolinite	Commerical (Sigma Adrich)	ML	< 200 µm	= 100	Kaolinite (~ 100%)	–
Montmorillonite	Commerical (Sigma Adrich)	CH	< 200 µm	= 100	Montmorillonite (~ 96%)	Quartz (~ 4%)

In order to characterize the chemical composition of tested soils, cation exchange capacity (CEC) and exchangeable cation measurements were completed on all soil materials using a process similar to U.S. EPA Method 9080^78^. During measurements, 10 g of dry soil and 50 mL of a 1 M NH_4_Cl solution were added to a plastic syringe and equilibrated for 12 h. Soil solutions were then extracted, collected, and exchangeable cations were characterized using ICP-MS. The remaining extracted soil samples were agitated in absolute ethanol for 6 h, decanted to remove ammonium (NH_4_^+^) ions that may have remained in free solution, and 50 mL of 1 M KCl solution was added to all samples and allowed to equilibrate for 12 h to encourage replacement of sorbed NH_4_^+^. Soil solutions were then extracted and NH_4_^+^ concentrations in the extracted solution were quantified using a salicylate colorimetric assay and used to calculate soil CEC values. Soil CEC values reflect the capacity of negatively charged soil surfaces to sorb cations and it was hypothesized that CEC differences between tested soils could affect ion exchange at soil surfaces and CaCO_3_ precipitation. Supplemental Table S1 presents both CEC values and exchangeable cations quantified for all soil materials used in this study.

### Cell culture preparation

*Sporosarcina pasteurii* (ATCC 11859) cell suspensions were prepared for batch experiments using frozen stock cultures and ATCC 1376 growth media (15.74 g/L tris base, 20 g/L yeast extract, 10 g/L ammonium sulfate, pH-adjusted to 9.0). All growth media volumes were autoclaved at 121 °C for 24 min, cooled to room temperature (23 °C), and inoculated with a *S. pasteurii* frozen stock culture. *S. pasteurii* stock cultures were prepared from freeze-dried cell pellets using ATCC 1376 growth media that was incubated, stabilized in 25% (v/v) glycerol, and stored at − 80 °C until use. Following inoculation, growth media volumes were incubated for 48 h at 30 °C using a double-orbital shaker at 175 rpm prior to harvesting cells. Concentrated *S. pasteurii* cell pellet suspensions were obtained by centrifuging ≈ 45 mL volumes of incubated growth media in a conical tube for 10 min at 1972 g, discarding the supernatant, rinsing the remaining cells using ≈ 45 mL of sterile isotonic saline solution (154 mM NaCl), mixing thoroughly, and repeating centrifuging and rinsing steps until the discarded supernatant appeared clear (≈ 2–3 rinse sequences). The cell rinsing process was performed both to concentrate cells and to remove growth factors present in ATCC 1376 growth media, which could have resulted in the growth of augmented cells during batch experiments, thereby impacting reaction progression and precipitation events. Final cell suspensions were prepared by adding 10 mL of sterile isotonic saline to rinsed cells and mixing thoroughly. The optical densities of these final cell suspensions were measured using a microplate spectrophotometer (Biotek Inc.) at a wavelength of 600 nm (OD_600_) and values typically ranged between 1.5 and 1.6, indicative of between 2.7 and 3.3 × 10^9^ cells/mL based upon a lab-specific total direct cell count to OD_600_ correlation (Supplemental Figure S1). Once diluted in cementation solutions, all experiments had estimated *S. pasteurii* cell densities between 6.6 and 8.2 × 10^7^ cells/mL, similar to augmented cell densities used in other bio-cementation experiments^25,29,79,80^.

### Treatment solutions

All treatment solutions were prepared using urea (Fisher Scientific Inc., > 99.2% assay), calcium chloride dihydrate (Fisher Scientific Inc., > 99.0% assay), and deionized water. Solutions contained 250 mM equimolar concentrations of urea and Ca^2+^ in all experiments with the exception of experimental series 3, which contained 250 mM urea, but minimal added Ca^2+^ (0 mM or 10 mM). Treatment solutions used for all controls were similar to solutions used in many past studies^36,45,81^ and contained only urea and Ca^2+^ to minimize potential effects from other additives. Since other solution compositions were not examined, however, the results of this study may be specific to these formulations. All other chemical constituents were added to solutions using dry reagents immediately prior to experiments including magnesium chloride hexahydrate (Fisher Scientific Inc., > 99.0% assay), strontium chloride hexahydrate (MP Biomedicals LLC, > 99.0% assay), sodium chloride (Fisher Scientific Inc., > 99.0% assay), and sodium sulfate (Fisher Scientific Inc., > 99.0% assay). Artificial seawater (ASW) solutions were prepared using S9983 dried sea salts (Millipore Sigma) wherein 100% ASW solutions contained 478 mM Na^+^, 536 mM Cl^−^, 54 mM Mg^2+^, 27 mM SO_4_^2−^, 10 mM Ca^2+^, 5 mM K^+^, 3 mM CO_3_^2−^, 0.1 mM Sr^2+^, and 0.03 mM B^3+^, which was consistent with average concentrations expected in natural seawater^82^. All treatment solutions were prepared more concentrated in order to achieve targeted concentrations after dilution with augmented soils, which contained ≈ 1 mL solution volumes from cell inoculants.

### Aqueous solution sampling

Small-volume aqueous solution samples (≈ 120 µL) were collected from all experiments at various times during reactions to monitor changes in solution urea and Ca^2+^ concentrations reflective of urea hydrolysis activity and CaCO_3_ precipitation. Sampling intervals varied between experiments due to differences in achieved ureolytic activities but included the collection of at least six samples over the first 10 h of experiments during which substantial reactant concentration changes were expected. In order to minimize the impact of sampling events on experimental behaviors, the total volume sampled for all experiments never exceeded 3.5% of the total solution volume (< 1.6 mL). All solution samples were collected near the center of plates using a pipette with disposable polypropylene tips. After collection, aqueous samples were pipetted into a 2.0 mL conical tube with a 0.22-µm cellulose acetate filter (Corning Inc.) and were centrifuged at 1318 g for a minimum of 30 s to remove solids. After filtration, 80 µL of filtered solution samples were added to 300 µL of a 1 M hydrochloric acid (Fisher Scientific) and samples were mixed thoroughly using a vortexer. Acid stabilization of samples was intended to mitigate potential volatilization of ammonia present within samples and the potential for continued ureolytic activity and CaCO_3_ precipitation reactions following sampling. All aqueous samples were frozen immediately after stabilization until thawing for subsequent urea and Ca^2+^ analyses.

### Aqueous chemical measurements

Measurements of aqueous urea and Ca^2+^ in time were performed for all samples obtained from batch experiments. Aqueous urea measurements were performed using a colorimetric assay modified from Knorst et al.^83^ wherein a colorimetric reagent consisting of 4% (w/v) p-Dimethylaminobenzaldehyde and 19% (v/v) HCl in absolute ethanol was added to dilute sample volumes. Absorbance values were measured at 422 nm using a microplate spectrophotometer and were compared to calibration curve relationships to determine sample concentrations. Aqueous Ca^2+^ measurements were completed using a QuantiChrom DICA-500 calcium assay kit (BioAssay Systems) with a phenolsulphonephthalein-based colorimetric dye. In this process, samples were first diluted in ultrapure water (< 7.2 × 10^−9^ mM Ca^2+^) to achieve concentrations within the linear range of the assay (< 5.0 mM Ca^2+^). 200 µL of a colorimetric reagent was then added to samples and absorbances were measured after 10 min at a wavelength of 612 nm. Supplemental Figure S2 provides example calibration curves for both assays used in this study.

### PHREEQC biogeochemical modeling

PHREEQC^84^, an open-source batch reaction aqueous geochemical code, was used to estimate cell densities for all experiments from observed urea degradation activity. In the PHREEQC model, microbial urea hydrolysis rates (ureolytic rates) were modeled using the cell-normalized Michaelis–Menten ureolytic kinetic expression presented in [Eq. 1] wherein ρ_cell_ is the *S. pasteurii* cell density in cells/L, K_m_ is the half-saturation coefficient in [mM urea] and V_max cell_ is the cell-normalized maximal velocity in [mM urea cell^−1^ h^−1^]^85^. In all models, K_m_ and V_max cell_ were assumed to be 305 mM urea and 1.057 × 10^−9^ mmol urea cell^−1^ h^−1^, respectively, following whole cell ureolytic parameters reported by Graddy et al.^86^ for *S. pasteurii* ATCC 11859.

1 $$\frac{{d\left[ {Urea} \right]}}{dt} = \left[ {\rho_{cell} } \right]\left( {\frac{{V_{\max cell} \left[ {Urea} \right]}}{{K_{m} + \left[ {Urea} \right]}}} \right)$$

In order to match modeled rates to experimentally observed urea degradation activity, *S. pasteurii* cell densities (ρ_cell_) in the models were varied. Recognizing that changes in microbial activity may occur during reactions due to a number of factors including cell death and encapsulation^43,87–89^, modeled trends were used to quantitatively estimate changes in ureolytic activity resulting from inhibitory ion concentrations and/or changes in cell viability during reaction periods. Since the primary motivation for calibration of the model to experimentally observed urea degradation data was to quantify ureolytic rates, CaCO_3_ precipitation was assumed to be an equilibrium reaction and all other kinetically controlled reactions were ignored. Cell density estimates from the PHREEQC model were compared to known augmented cell densities measured using OD_600_ measurements to evaluate consistency between activity-based estimates and direct cell density measurements. Initial urea degradation rates were also determined for experiments using the cell-normalized Michaelis–Menten ureolytic kinetic model, estimated cell densities, and the known initial urea concentration of 250 mM.

### X-ray diffraction analyses

X-ray diffraction (XRD) analyses were performed to characterize the mineralogy of CaCO_3_ precipitates from batch experiments, identify soil minerals, and detect the presence of any other non-CaCO_3_ minerals that may have formed. Characterization of CaCO_3_ precipitates specifically focused on detecting and quantifying the presence of calcite, vaterite, and aragonite, all of which are crystalline CaCO_3_ polymorphs. All XRD analyses were performed using a Bruker D8 Discover X-ray powder diffractometer with a IμS 2-D powder microfocus source, a high-efficiency Cu anode, and a Pilatus 100 k large-area 2-D detector. Scans were performed using a phi rotation of 45 s for 2θ values from 10.6° to 99.4° at increments of 0.02°. Samples were prepared from absolute ethanol-rinsed precipitates obtained from batch experiments and were ground into a fine powder using an agate mortar and pestle prior to XRD analyses in order to obtain particle sizes between ≈ 10 and 50 µm following recommendations by Pecharsky and Zavalij^90^. Semi-quantitative weight percentage analysis (S-Q analysis) of the obtained diffraction patterns were performed to estimate relative quantities (by mass) of the different CaCO_3_ mineral phases present using Diffrac.EVA XRD software (version 4.3) and the ICDD PDF2, ICDD PDF4, and COD reference diffraction databases^91^. S-Q analyses involve the fitting of measured diffraction patterns using integrated diffraction peak intensities for known crystalline minerals, in order to determine the presence of minerals and their relative fractions by mass. Relative CaCO_3_ percentages were determined from S-Q analyses for aragonite, vaterite, and calcite minerals, specifically, and had an estimated error of ± 5% by mass which was determined from independent characterizations of select specimens using Fourier transform infrared spectroscopy (FTIR). Table 3 summarizes S-Q analysis results for all experiments. Since x-ray diffraction requires the knowledge of known refraction angles for crystalline atomic structures, the performed XRD analyses were unable to reliably quantify amorphous CaCO_3_.

**Table 3:** Summary of X-ray diffraction (XRD) S-Q analysis results for all precipitates.

Specimen name	Experimental series	Parent soil	Relative % of CaCO_3_ (By Mass)*			Other detected minerals (in order of decreasing abundance)
			% Calcite	% Vaterite	% Aragonite	
E1_0% ASW	(1) ASW	Ottawa F-65 sand	85	9	6	Quartz
E2_50% ASW	(1) ASW	Ottawa F-65 sand	81	10	9	Quartz
E3_100% ASW	(1) ASW	Ottawa F-65 sand	80	7	13	Quartz
E4_0 mM Mg^2+^/0 mM Sr^2+^	(2) ASW ions	Ottawa F-65 sand	86	8	6	Quartz
E5_27 mM Mg^2+^	(2) ASW ions	Ottawa F-65 sand	85	9	6	Quartz
E6_54 mM Mg^2+^	(2) ASW ions	Ottawa F-65 sand	90	5	5	Quartz
E7_108 mM Mg^2+^	(2) ASW ions	Ottawa F-65 sand	81	11	8	Quartz, Magnesian Calcite
E8_0.055 mM Sr^2+^	(2) ASW ions	Ottawa F-65 sand	88	6	6	Quartz
E9_0.11 mM Sr^2+^	(2) ASW ions	Ottawa F-65 sand	86	8	6	Quartz
E10_0.22 mM Sr^2+^	(2) ASW ions	Ottawa F-65 sand	85	9	6	Quartz
E11_14 mM SO_4_^2−^	(2) ASW ions	Ottawa F-65 sand	85	10	5	Quartz
E25_Ottawa sand	(5) Soil variations	Ottawa F-65 sand	85	10	5	Quartz
E26_Fraser river sand	(5) Soil variations	Fraser river sand	86	7	7	Quartz, Albite
E27_Concrete sand	(5) Soil variations	Concrete sand	92	5	3	Quartz, Albite
E28_Covelo sand	(5) Soil variations	Covelo sand	91	5	4	Quartz, Albite
E29_Delta sand	(5) Soil variations	Delta sand	89	7	4	Quartz, Albite
E30_Monterey sand	(5) Soil variations	Monterey sand	86	8	6	Quartz, Microcline, Albite
E31_Feldspar	(5) Soil variations	Feldspar	87	7	6	Microcline, Albite, Quartz
E32_Olivine	(5) Soil variations	Olivine	93	4	3	Forsterite, Fayalite
E33_Mica	(5) Soil variations	Mica	88	9	3	Lepidolite
E34_Kaolinite	(5) Soil variations	Kaolinite	86	10	4	Kaolinite
E35_Montmorillonite	(5) Soil variations	Montmorillonite	80	14	6	Montmorillonite, Quartz

*XRD S-Q analyses have an estimated error of ± 5% by mass following other independent analyses.

### Scanning electron microscope imaging

Scanning electron microscope (SEM) imaging was completed to examine the morphology of CaCO_3_ precipitates including the relative size, shape, and distribution of CaCO_3_ crystals within specimens. SEM imaging was performed using a FEI XL830 dual-beam focused ion beam scanning electron microscope using acceleration voltages between 1 and 5 kV and magnifications between 200x and 1000x. Prior to imaging, precipitates were oven-dried at 110 °C for at least 2 days, subsampled, mounted to imaging pedestals using carbon tape, and sputter-coated using a 60%/40% Au/Pd alloy target for 60 s at a deposition rate of 13 nm/min in an argon gas chamber. Sputter-coating was completed to increase the conductivity of samples, minimize charging effects, and improve image resolution^92^.

### Precipitate chemical composition analyses

For select experiments, CaCO_3_ precipitates were dissolved in dilute acid solutions to further examine their chemical composition. Prior to the acid dissolution process, dry precipitate subsamples (≈ 1 g) were rinsed and decanted three times using absolute ethanol to remove soluble ions, subsamples were oven-dried at 110 °C for at least 2 days, and 0.5-g subsamples were added to 25 mL of a 15 mM hydrochloric acid solution to induce dissolution. Samples were allowed to equilibrate for 72 h, during which samples were mechanically agitated once every 24 h. Resulting aqueous solutions, which contained ions from dissolved precipitates, were filtered using 0.45-µm syringe filters to remove solid particulate and select ion concentrations were quantified using ICP-MS measurements. Supplemental Table S2 provides a full summary of aqueous ion concentrations present in dissolution solutions. Ca^2+^ concentrations in solution samples ranged between 3.8 mM and 5.5 mM and were consistent with concentrations observed in similar dissolution experiments^93^. Ratios between various ion concentrations and Ca^2+^ concentrations were examined to further evaluate incorporation and substitution of Ca^2+^ ions in precipitates.

## Results and discussion

### Artificial seawater experiments

**Figure 1:**
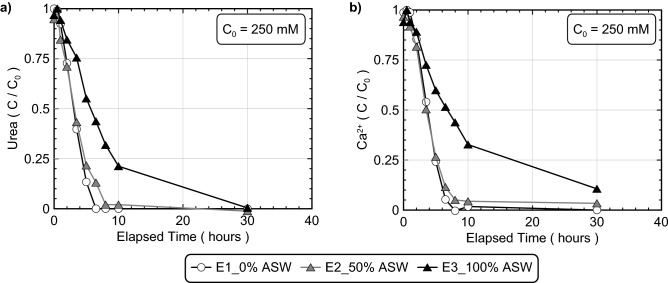
Normalized concentrations of (**a**) urea and (**b**) Ca^2+^ in time for 0%, 50%, and 100% ASW experiments. All experiments included the addition of 250 mM urea, 250 mM Ca^2+^, *S. pasteurii* cells at 7.6 × 10^7^ cells/mL, and varying ASW additions.

Figure 1 presents normalized concentrations of urea and Ca^2+^ in time for artificial seawater experiments (experimental series 1). All experiments contained *S. pasteurii* cells at a cell density of 7.6 × 10^7^ cells/mL and 250 mM urea and Ca^2+^ to initiate bio-cementation in a deionized water-based solution with varying concentrations of artificial seawater (ASW) by volume, representative of freshwater (0% ASW), brackish (50% ASW), and seawater (100% ASW) geochemical environments. As shown in Fig. 1a, both the 0% and 50% ASW experiments showed similar urea degradation behaviors, however, the 100% ASW experiment had an initial urea degradation rate that was nearly ≈ 50% lower than that of the 0% ASW control specimen. This activity reduction likely resulted from the higher ionic strength and cation (i.e., Na^+^, Mg^2+^, SO_4_^2−^, Ca^2+^, K^+^, Sr^2+^, B^3+^) concentrations present in seawater, which have been previously shown to interfere with intercellular diffusion and enzyme activity across a broad range of bacterial species^94,95^ as well as inhibit ureolysis in select indigenous ureolytic microorganisms^96^. When considering Ca^2+^ concentrations in time shown in Fig. 1b, significantly slower precipitation rates were also observed in the 100% ASW experiment, similar to urea degradation trends. The agreement between urea and Ca^2+^ concentration trends in time suggested that CaCO_3_ precipitation rates were limited by urea hydrolysis activity, with CaCO_3_ precipitation occurring exceedingly fast following the availability of CO_3_^2−^ from urea degradation.

**Figure 2:**
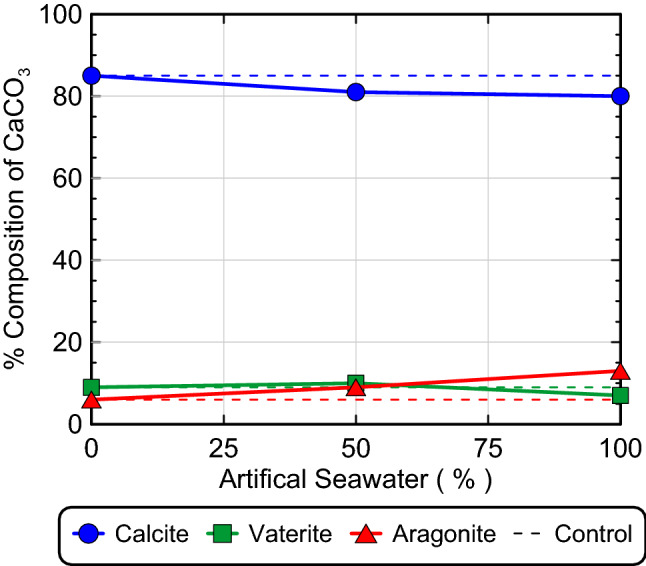
Relative CaCO_3_ mineral content percentages for 0%, 50%, and 100% ASW experiments determined from S-Q analysis of XRD diffraction patterns.

Figure 2 presents results from S-Q analyses completed on XRD diffraction patterns obtained from the ASW precipitate samples. As shown, calcite was the dominant mineral phase in all experiments (> 80%), with minor decreases in calcite relative percentages (≈ 5%) and minor increases in aragonite relative percentages as ASW was increased from 0 to 100% by volume. In the 100% ASW experiment, XRD diffraction patterns corresponding to calcite exhibited a small 2θ shift of ≈ 0.2° consistent with the reference signal for magnesian calcite, a mineral wherein Mg^2+^ is partially substituted for Ca^2+^ within the crystalline structure of calcite^97^. Although magnesite (MgCO_3_) was expected to form in ASW specimens due to its relatively low solubility (K_sp_ = 10^−8.03^), no magnesite peaks were detected in any of the obtained diffraction patterns for the ASW experiments^98^. Since calcite (K_sp_ = 10^−8.48^) has a lower solubility than magnesite, it remains possible that some magnesite may have precipitated during the treatment process but dissolved near the end of reactions due to reductions in surrounding aqueous CO_3_^2−^ activities^98^.

**Figure 3:**
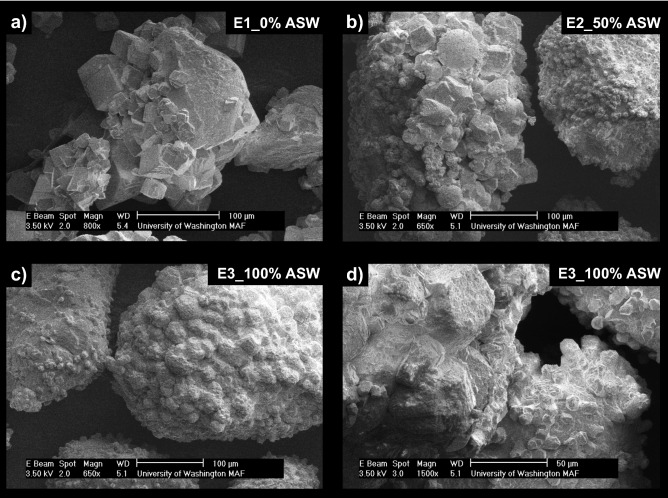
SEM images of precipitates from (**a**) 0% ASW, (**b**) 50% ASW, and (**c, d**) 100% ASW experiments.

Although XRD analyses indicated that minimal changes in precipitate mineralogy occurred with changes in ASW concentrations, SEM images of the ASW experiments (Fig. 3) exhibited more pronounced changes in precipitate morphology. As shown in Fig. 3a, the 0% ASW control specimen exhibited almost exclusively rhombohedral crystal morphologies characteristic of calcite, however, the 50% ASW specimen (Fig. 3b) included spherical vaterite-like morphologies that were interspersed with rhombohedral crystal forms. Many of the rhombohedral crystal forms present in the 50% ASW specimen also exhibited duller, more well-rounded edges in comparison to the sharper, more well-defined crystalline structures observed in the 0% ASW control. In the 100% ASW specimen, morphological differences were even more pronounced (Fig. 3c,d), with increases in the abundance more well-rounded rhombohedral crystals and crystal surfaces which appeared to have a rougher appearance in comparison to the smoother crystal faces found in the 0% ASW control.

### Discrete seawater ion experiments

**Figure 4:**
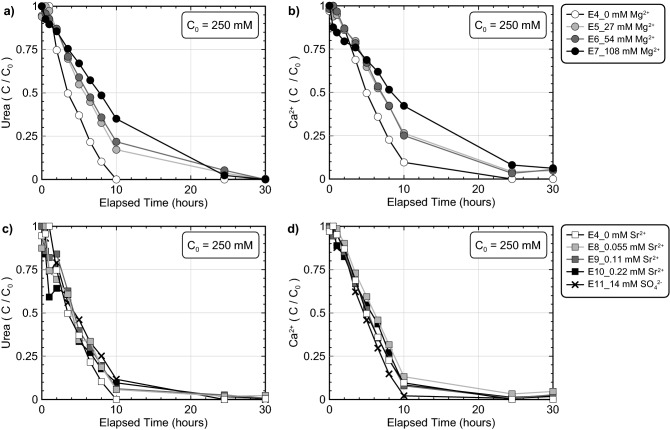
Normalized concentrations of (**a, c**) urea and (**b, d**) Ca^2+^ in time for (**a, b**) Mg^2+^ varied experiments and (**c, d**) Sr^2+^ and SO_4_^−2^ varied experiments. All experiments included the addition of 250 mM urea, 250 mM Ca^2+^, *S. pasteurii* cells at 7.1 × 10^7^ cells/mL, and varying Mg^2+^, Sr^2+^, or SO_4_^2−^ additions.

Although the previous experiments demonstrated that seawater ions could influence bio-cementation morphology and reaction kinetics, it remained unclear which specific ions were responsible for these differences. A series of experiments were therefore performed which included varying concentrations of Mg^2+^, Sr^2+^, and SO_4_^2−^ in order to further investigate the effect of discrete seawater ions additions (experiment series 2). Experiments were designed to examine the effect of ions at concentrations corresponding to ≈ 50%, ≈ 100%, and ≈ 200% of their expected concentrations in seawater, with the exception of SO_4_^2−^, which could not be prepared at concentrations exceeding 14 mM (≈ 50% ASW) due to the immediate precipitation of gypsum (CaSO_4_) in cementation solutions. All experiments contained *S. pasteurii* cells at a cell density of 7.1 × 10^7^ cells/mL, 250 mM urea and Ca^2+^ to initiate bio-cementation, and varying concentrations of ASW ions. Figure 4 presents normalized concentrations of urea and Ca^2+^ in time for all discrete seawater ion experiments including those with Mg^2+^ variations (Fig. 4a,b) and SO_4_^2−^ and Sr^2+^ variations (Fig. 4c,d). As shown, increases in Mg^2+^ concentrations resulted in progressive inhibition of ureolytic rates (Fig. 4a) with a ≈ 15% decrease in initial ureolytic rates observed in the 27 mM and 54 mM experiments and a larger ≈ 45% decrease in initial ureolytic rate observed in the 108 mM Mg^2+^ experiment. In contrast, when Sr^2+^ and SO_4_^2−^ were present at concentrations up to 0.22 mM and 14 mM, respectively, no substantial differences in ureolytic rates were observed (Fig. 4c). Despite the presence of additional cations, urea and Ca^2+^ trends in time were again similar with slightly elevated Ca^2+^ concentrations observed in the Mg^2+^ varied experiments (Fig. 4b). Although minimal inhibition of CaCO_3_ precipitation was observed in the 14 mM SO_4_^2−^ experiment, greater inhibition of precipitation may have been observed if higher SO_4_^2−^ concentrations near 24 mM were present, as noted by Busenberg and Plummer^65^.

**Figure 5:**
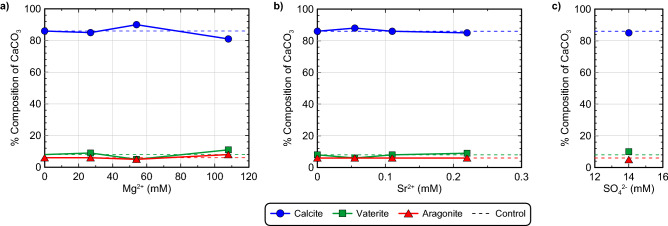
Relative CaCO_3_ mineral content percentages for (**a**) Mg^2+^ varied experiments, (**b**) Sr^2+^ varied experiments, and the (**c**) 14 mM SO_4_^2−^ experiment determined from S-Q analysis of XRD diffraction patterns.

Figure 5 presents the results of S-Q analyses completed on the XRD diffraction patterns obtained from precipitate samples for all discrete seawater ion experiments. Again, calcite was the predominant mineral phase with relative percentages exceeding 81% in all experiments. At the highest Mg^2+^ concentration considered (108 mM), a slight decrease in the relative percentage of calcite (≈ 5%) was observed with a corresponding increase in vaterite and aragonite (Fig. 5a). Similar to the previous ASW experiments, XRD diffraction patterns again indicated the presence of magnesian calcite in Mg^2+^ amended experiments, although its relative abundance was sufficiently small that it could not be reliably quantified (< 2% by mass). No other Mg^2+^ containing minerals (e.g., MgCO_3_) were detected in precipitates. When Sr^2+^ concentrations were increased and 14 mM SO_4_^2−^ was added, no substantial deviations in mineralogical compositions were observed from the control specimen (Fig. 5b,c). No other non-CaCO_3_ mineral phases were detected in the obtained precipitates.

**Figure 6:**
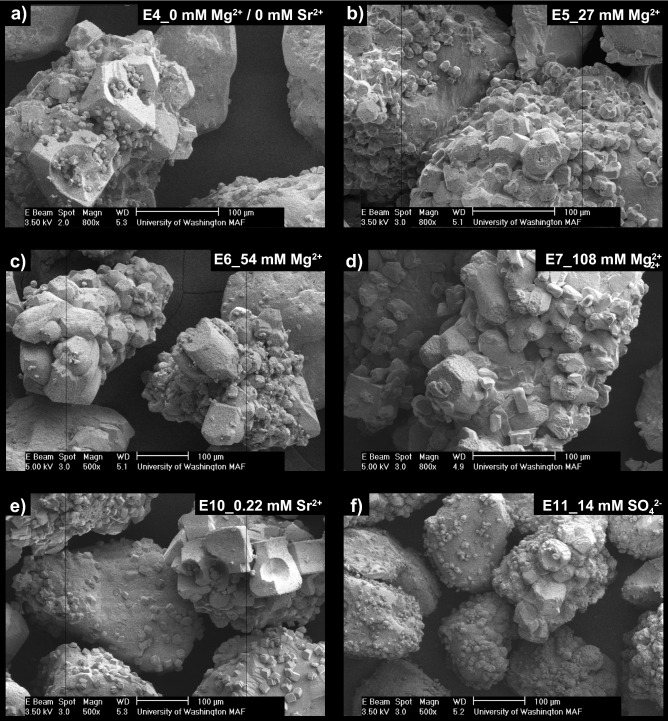
SEM images of precipitates from the (**a**) 0 mM Mg^2+^/0 mM Sr^2+^, (**b**) 27 mM Mg^2+^, (**c**) 54 mM Mg^2+^, (**d**) 108 mM Mg^2+^, (**e**) 0.22 mM Sr^2+^ and (**f**) 14 mM SO_4_^2−^ experiments.

SEM images of precipitates again revealed more dramatic differences between experiments (Fig. 6). In the deionized water control (Fig. 6a), rhombohedral crystals were observed that were consistent with calcite and morphologies found in the previous control (Fig. 3a). Small spherical voids in rhombohedral crystals, consistent with the shape of vaterite crystals, were also occasionally observed in the control. Similar morphologies have been observed in previous abiotic studies and have been attributed to the dissolution and re-precipitation of vaterite via Ostwald ripening^99^. In this process, some vaterite crystals that are precipitated initially under highly supersaturated conditions, dissolve to form lower solubility calcite crystals near the end of reactions, thereby leaving behind calcite crystals with vaterite-like “casts” or impressions^100^. In the 27 mM Mg^2+^ experiment, crystal morphologies were further modified and were more similar to dodecahedrons (Fig. 6b) which also displayed a greater degree of anisotropy when compared to the control. Similar crystal forms were also observed at higher Mg^2+^ concentrations (Fig. 6c,d), with crystal surfaces becoming rougher and crystal edges becoming duller in appearance as Mg^2+^ concentrations increased. Crystal morphologies resembling cylinders were also prominent in the 108 mM Mg^2+^ experiment (Fig. 6c,d). Interestingly, morphologies in the Mg^2+^ experiments differed from those previously observed in the ASW experiments with spherical forms consistent with vaterite being largely absent. When considering the effect of Sr^2+^ and SO_4_^2−^ additions on precipitate morphology, both the highest concentration 0.22 mM Sr^2+^ and 14 mM SO_4_^2−^ specimens had well-defined rhombohedral crystals, which did not substantially differ from the control experiment (Fig. 6e,f).

### Discrete seawater ion experiments (ureolysis)

**Figure 7:**
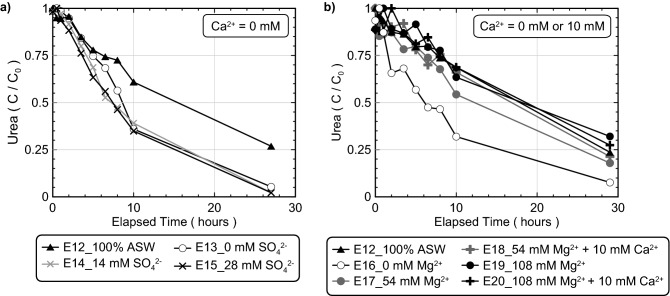
Normalized concentrations of urea in time for experiments with (**a**) varying SO_4_^2−^ and ASW concentrations and no added Ca^2+^ and (**b**) varying Mg^2+^ and ASW concentrations with minimal added Ca^2+^. All experiments included the addition of 250 mM urea, 0 mM or 10 mM Ca^2+^, *S. pasteurii* cells at 6.6 × 10^7^ cells/mL, and varying SO_4_^2−^, Mg^2+^, or ASW additions.

While earlier experiments evaluated the effect of discrete seawater ions on bio-cementation morphology, mineralogy, and reaction kinetics, the primary mechanisms responsible for ureolytic inhibition the high Mg^2+^ and 100% ASW experiments remained unclear. It was hypothesized that the higher Mg^2+^ concentrations supplied in these experiments may have interfered with cellular and enzymatic activity directly or resulted in the formation of additional precipitates during the reaction period that could have inhibited ureolytic activity via cell encapsulation. In addition, while Sr^2+^ and SO_4_^2−^ additions were shown to have minimal effects on process kinetics, the effect of SO_4_^2−^ on ureolytic activity was not explored at concentrations present in 100% ASW due to solution preparation limitations. In order to better isolate the effect of SO_4_^2−^ and Mg^2+^ additions on ureolytic activity alone, a series of experiments were performed containing minimal Ca^2+^ intended to eliminate CaCO_3_ precipitation and its related effects on process kinetics (experimental series 3). Figure 7 presents normalized concentrations of urea in time for all experiments containing minimal Ca^2+^ including those with SO_4_^2−^ and ASW (Fig. 7a) and Mg^2+^ and ASW (Fig. 7b). As shown in Fig. 7a, SO_4_^2−^ concentrations comparable to that present in 100% ASW (≈ 28 mM) had no detectable effects on urea hydrolysis activity. In contrast, urea hydrolysis in 100% ASW was again significantly slower than the deionized water controls (E13, E16) despite containing minimal Ca^2+^ (10 mM) when compared to the earlier 100% ASW experiment that was augmented with 250 mM Ca^2+^. Figure 7b presents normalized concentrations of urea in time for Mg^2+^ varied experiments containing either 10 mM Ca^2+^ or no added Ca^2+^ (0 mM) along with 100% ASW and deionized water control experiments. The addition of 10 mM Ca^2+^ was included in select experiments to match the Ca^2+^ present in 100% ASW. For some specimens, Ca^2+^ additions were also entirely removed to mitigate any potential inhibitory effects related to CaCO_3_ precipitation. As shown, for all experiments containing more than 54 mM Mg^2+^, ureolytic activity was significantly inhibited when compared to the control regardless of the supplied Ca^2+^ concentration. When comparing ureolytic activity in the 54 mM Mg^2+^ with 10 mM Ca^2+^ experiment to the 100% ASW specimen, however, ureolytic rates were nearly identical, suggesting that the inhibition of ureolysis observed in 100% ASW may primarily result from the presence of 54 mM Mg^2+^.

### Sodium varied experiments

**Figure 8:**
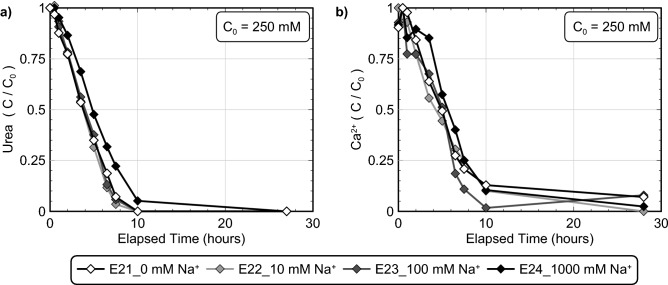
Normalized concentrations of (**a**) urea and (**b**) Ca^2+^ in time for Na^+^ varied experiments. All experiments included the addition of 250 mM urea, 250 mM Ca^2+^, *S. pasteurii* cells at 8.0 × 10^7^ cells/mL and varying added Na^+^ concentrations.

**Figure 9:**
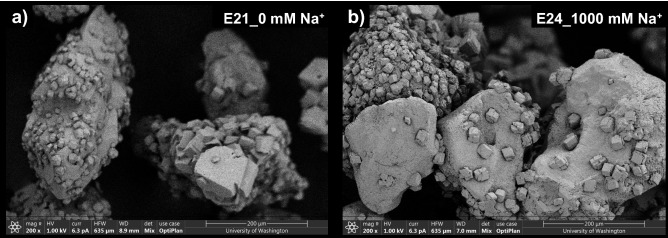
SEM images of precipitates from (**a**) 0 mM Na^+^ and (**b**) 1000 mM Na^+^ specimens.

In the previous experiments which examined the effect of ASW and discrete seawater ion concentrations, the effect of corresponding ionic strength increases remained relatively unknown. A series of experiments were therefore performed to examine the effect of solution ionic strength differences as realized by NaCl additions (experiment series 4). NaCl additions were used in these experiments to examine ionic strength changes due to their frequent use as an inert electrolyte in biological experiments^101^. All experiments contained *S. pasteurii* cells at a cell density of 8.0 × 10^7^ cells/mL, 250 mM urea and Ca^2+^ to initiate bio-cementation, and either 0 mM (I ≈ 753 mM), 10 mM (I ≈ 763 mM), 100 mM (I ≈ 853 mM), or 1000 mM Na^+^ (I ≈ 1753 mM). Figure 8 presents normalized concentrations of urea and Ca^2+^ in time for all Na^+^ varied experiments. As shown, no detectable changes in ureolytic rates were observed at Na^+^ concentrations up to 100 mM. In the 1000 mM Na^+^ experiment, which had an ionic strength nearly twice as large, however, initial ureolytic rates were reduced by ≈ 20% when compared to the control. Although ureolytic inhibition observed in the 100% ASW experiments were primarily attributed to the 54 mM Mg^2+^ concentrations, the ionic strength of the 100% ASW solutions were near 1458 mM when 250 mM Ca^2+^ was supplied. Thus, it is likely that some smaller fraction of the ureolytic inhibition observed in 100% ASW may be related to the higher ionic strength of these solutions. Figure 9 presents SEM images of precipitates from both the 0 mM and 1000 mM Na^+^ experiments. As shown, precipitates from both the control (Fig. 9a) and 1000 mM Na^+^ experiment (Fig. 9b) exhibited consistent rhombohedral calcite-like crystals and no other substantial differences in precipitate morphology, distribution, or size were observed as a function of Na^+^ variations.

### Soil varied experiments

**Figure 10:**
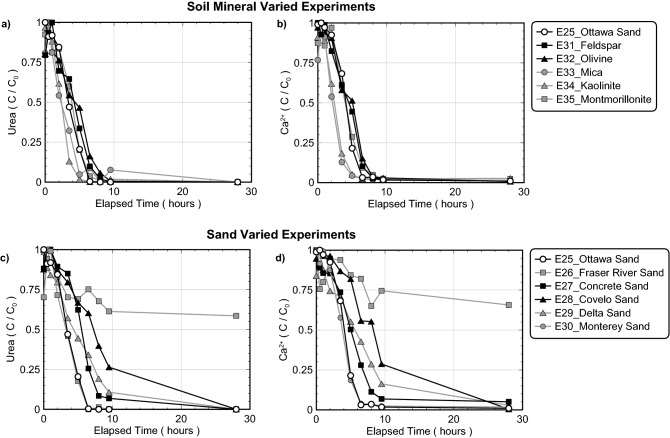
Normalized concentrations of (**a, c**) urea and (**b, d**) Ca^2+^ in time for (**a, b**) soil mineral varied experiments and (**c, d**) poorly-graded sand varied experiments. All experiments included the addition of 250 mM urea, 250 mM Ca^2+^, *S. pasteurii* cells at 8.2 × 10^7^ cells/mL, and varying soil materials.

In order to investigate the effect of changes in soil materials, a series of experiments were performed using eleven different soils (experiment series 5). Figure 10 presents normalized concentrations of urea and Ca^2+^ in time for all soil varied experiments including relatively pure soil minerals (Fig. 10a,b) and natural and processed poorly-graded sands (Fig. 10c,d). All experiments contained *S. pasteurii* at a cell density of 8.2 × 10^7^ cells/mL, identical treatment solutions containing 250 mM urea and Ca^2+^ to initiate bio-cementation, but different soils. As shown in Fig. 10a, minimal changes in ureolytic rates were observed between experiments containing different pure soil minerals with near full urea hydrolysis occurring after 10 h. Although unexpected, the kaolinite and mica specimens exhibited slightly faster initial ureolytic rates when compared to the quartz-based Ottawa F-65 Sand control, which may have resulted from the hydration of these minerals, local increases in urea concentrations, and small increases in ureolytic rates. In other experiments containing different poorly-graded sands (Fig. 10c), however, more significant variations in ureolytic rates were observed when compared to the control. In particular, the Fraser River Sand experiment exhibited strong inhibition of ureolysis within the first 10 h, with urea degradation ceasing entirely after only ≈ 35% reaction completion. Although not as pronounced, minor reductions in ureolytic rates were also observed in the Covelo, Delta, and Concrete Sand experiments. While all considered sands were primarily composed of quartz and feldspar minerals, significant differences in exchangeable cation concentrations were detected between these materials (Supplemental Table S1). Notably, the Fraser River Sand material contained the highest concentrations of exchangeable Al^3+^, Zn^2+^, and Fe^3+^ of all considered sands. In addition, all sands exhibiting detectable ureolytic inhibition (Covelo, Delta, Concrete, and Fraser River Sand) contained higher concentrations of exchangeable Mg^2+^ (60–239 µg/g soil) and barium (Ba^+^) (5–21 µg/g soil) when compared to Ottawa F-65 Sand and Monterey Sand, which had similar ureolytic activities and much lower Mg^2+^ and Ba^+^ concentrations (Mg^2+^  =  < 0.2–31 µg/g soil, Ba^+^  = 0.2–0.3 µg/g soil). Although the observed ureolytic rate differences could not be definitively attributed to these specific ions, these results do suggest that differences in exchangeable soil ions may significantly influence the ureolytic activity of augmented bacteria and should be characterized when assessing the treatment feasibility of new soils. When comparing urea and Ca^2+^ trends in time from all soil varied experiments, similar behaviors were again observed, with the Fraser River Sand specimen achieving limited utilization of the supplied Ca^2+^ due to the inhibition of ureolysis.

**Figure 11:**
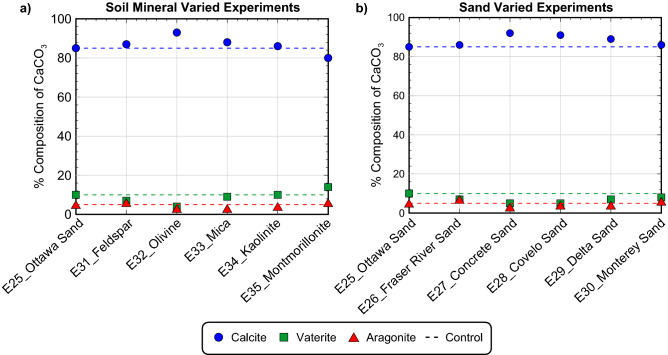
Relative CaCO_3_ mineral content percentages for (**a**) soil mineral varied experiments and (**b**) poorly-graded sand varied experiments determined from S-Q analysis of XRD diffraction patterns.

Figure 11 presents the results of S-Q analyses completed on the XRD diffraction patterns obtained from precipitate samples for all soil varied experiments. As shown, calcite was again found to be the dominant mineral phase with relative percentages exceeding 80%. The montmorillonite experiment had the lowest calcite relative percentage of 80% which was ≈ 5% less than the Ottawa F-65 Sand control as well as the highest relative percentage of vaterite at ≈ 14%. All other experiments had relative calcite percentages between ≈ 85% and 93%, vaterite percentages between ≈ 4% and 10%, and aragonite percentages between ≈ 3% and 6%, and were similar to the control. Collectively, these results suggested that changes in soil materials had minimal effects on the mineralogy of resulting precipitates, despite having more pronounced impacts on reaction kinetics.

**Figure 12:**
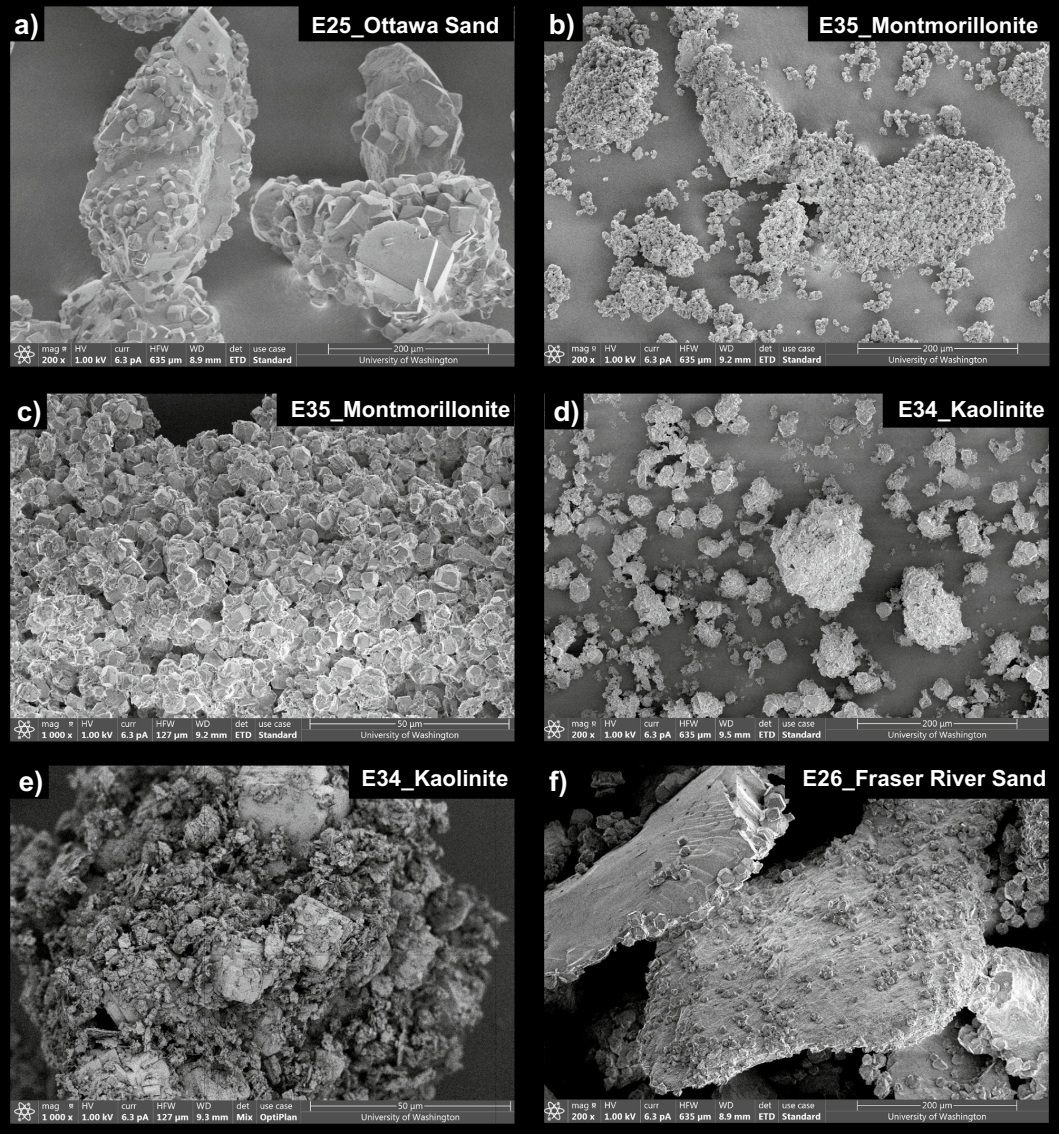
SEM images of precipitates from (**a**) Ottawa F-65 Sand, (**b, c**) montmorillonite, (**d, e**) kaolinite, and (**f**) Fraser River Sand experiments.

Figure 12 presents SEM images of precipitates from select soil varied experiments. As shown in Fig. 12a, precipitation in the Ottawa F-65 Sand control exhibited rhombohedral morphologies consistent with those expected for calcite and other control experiments. Although morphological differences between experiments were more difficult to assess given differences in soil particle sizes and geometries, precipitation in the montmorillonite experiment appeared to consist of rhombohedral crystals that were consistently smaller (diameter ≈ 5 µm) than those present in the control (diameter ≈ 10 to 50 µm) (Fig. 12b, c). CaCO_3_ crystals also appeared to form large clusters of loosely bound precipitates in the montmorillonite specimen, an outcome which was unexpected given the much larger specific surface area of this soil. Although XRD analyses suggested that slightly higher vaterite relative percentages were present in the montmorillonite experiment, clear morphological differences were not observed. In the kaolinite experiment, rhombohedral crystals consistent with calcite were interspersed with clay particles (Fig. 12d,e) and appeared to be larger than those in the montmorillonite specimen. This was consistent with earlier XRD analyses, which suggested that the mineralogical composition of the kaolinite experiment was similar to that of the control. Lastly, SEM images of the Fraser River Sand experiment (Fig. 12f) exhibited morphologies consistent with calcite with no discernable morphological differences when compared to the control. While this specimen exhibited significant inhibition of ureolysis, this outcome did not appear to significantly influence resulting CaCO_3_ precipitation mineralogy or morphology.

### Comparison of calcium utilization and urea degradation

**Figure 13:**
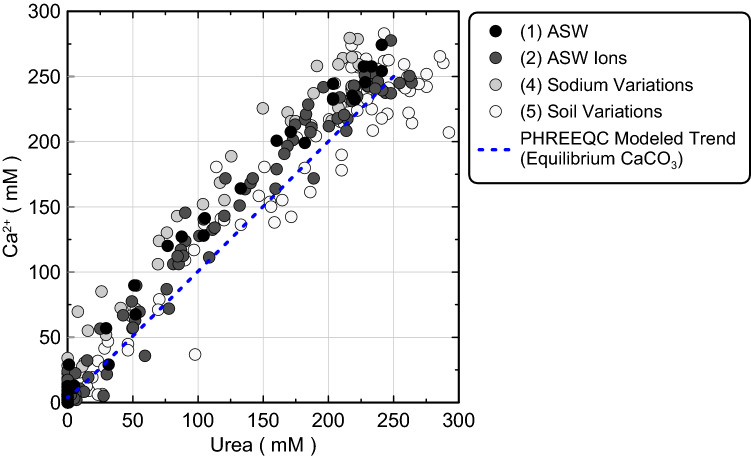
Corresponding measurements of aqueous Ca^2+^ and urea concentrations from all experiments as well as a PHREEQC modeled trend which assumes CaCO_3_ precipitation to be an equilibrium reaction.

Figure 13 presents corresponding aqueous Ca^2+^ and urea concentration measurements for all experiments during bio-cementation as well as a PHREEQC modeled trend, which assumed CaCO_3_ precipitation to be an equilibrium reaction (immediate precipitation of CaCO_3_ upon supersaturation of solutions). As shown, all aqueous Ca^2+^ and urea concentrations had initial values near 250 mM and followed a near 1:1 slope as precipitation reactions proceeded toward completion. Ca^2+^ concentrations were generally between 0 and 40 mM higher than corresponding urea concentrations, however, suggesting that CaCO_3_ precipitation was limited by urea hydrolysis as expected. When comparing Ca^2+^ and urea concentration measurements to the PHREEQC modeled trend, measurements and modeled values exhibited good agreement suggesting that CaCO_3_ precipitation may occur relatively quickly following CO_3_^2−^ availability from urea hydrolysis with the kinetics of CaCO_3_ precipitation being reasonably approximated as an equilibrium reaction. Despite variations in soil materials and geochemical conditions between the performed experiments, no substantial differences in trends were observed. The agreement between Ca^2+^ and urea concentration measurements and the modeled equilibrium reaction trend is consistent with other recent reactive transport modeling efforts^102,103^, which have suggested that the kinetics of calcite precipitation can be well approximated as an equilibrium reaction. This outcome suggests that more other computationally intensive CaCO_3_ precipitation kinetic expressions such as those related to changes in mineral specific surface areas and saturation state^104–107^ used by previous researchers^108–111^ may be avoided when attempting to geochemically model MICP applications when conditions are similar to those considered in this study.

### Comparison of OD_600_ and PHREEQC-based cell density estimates

For all experiments, augmented cell densities were quantified in two ways: (1) OD_600_ measurements with conversion to total cell densities using a OD_600_ to total direct cell count conversion, and (2) calibration of the cell-normalized Michaelis–Menten ureolytic kinetic expression (Eq. 1) to experimentally observed urea degradation behaviors using whole cell enzymatic parameters for *S. pasteurii* from Graddy et al.^86^ and PHREEQC batch reaction models. The kinetic model was calibrated to observed ureolytic activity: (1) to assess the ability of known augmented cell densities and whole cell enzymatic parameters to accurately estimate ureolytic activities across a broad range of experimental conditions and (2) to quantify differences in ureolytic activities between experiments as a function of environmental factors. In order to examine the former, the ratio of cell densities estimated using the PHREEQC model to known augmented cell densities from OD_600_ measurements were compared between all experiments (Table 1, Supplemental Figure S3). Significant discrepancies were observed between activity-based cell density estimates and known augmented cell densities with PHREEQC activity-based estimates ranging between 9 and 208% of known directly measured values. As expected, PHREEQC activity-based estimates substantially underestimated augmented cell densities for experiments exhibiting significant ureolytic inhibition including the 100% ASW (E03), high Mg^2+^ (E05–E07), and Fraser River Sand (E26) specimens. Surprisingly, considerable variations were also observed for control experiments wherein PHREEQC estimates varied between 68 and 171% of known augmented values. Although these results are specific to the whole cell enzymatic parameters^86^, experimental conditions, and cell preparation methods used in this study, results clearly demonstrated that augmented per cell activities vary significantly with differences in environmental conditions and should be carefully considered when attempting to model ureolytic activity for MICP field applications. From this perspective, direct measurements of ureolytic activity via urea concentration measurements or other means may afford superior insights when compared to the knowledge of augmented cell densities alone.

### Comparison of ureolytic activity

In order to better quantify differences in ureolytic activity between experiments under varying environmental conditions and respective control experiments, PHREEQC activity-based cell density estimates for experiments were also compared to the PHREEQC activity-based cell density estimates obtained for control experiments from the same experimental series (Supplemental Figure S4) to identify conditions which most significantly affected ureolytic activity. Although all control experiments had similar ureolytic activities, small variations in augmented cell densities and per cell activities between cell batches resulted in some differences between controls from different experimental series. When comparing activities, PHREEQC cell density estimates for all non-control experiments varied between 6 and 131% of their respective controls. Of these experiments, ureolytic inhibition was the most significant for the Fraser River Sand (6%) and 108 mM Mg^2+^ experiments (33%) with acceleration of ureolysis occurring in only the mica (131%) and montmorillonite (127%) experiments. Collectively, these results highlight five primary outcomes: (1) increases in ASW concentrations progressively decreased ureolytic activities, (2) increases in Mg^2+^ concentrations resulted in increased inhibition of ureolysis both with and without Ca^2+^ present, (3) SO_4_^2−^ and Sr^2+^ had no detectable effects on ureolysis at concentrations up to twice that present in seawater, (4) increases in ionic strength via NaCl additions resulted in no detectable effects on ureolysis at low concentrations (< 100 mM Na^+^), but detectable inhibition at higher concentrations (1000 mM Na^+^) which corresponded to an increase in ionic strength near one order of magnitude similar to that present in 100% ASW, and (5) variations in soil materials almost always resulted in decreased ureolytic activity relative to the Ottawa F-65 Sand control, which may be attributed to the presence of inhibitory exchangeable soil ions.

### Precipitate composition analyses

**Figure 14:**
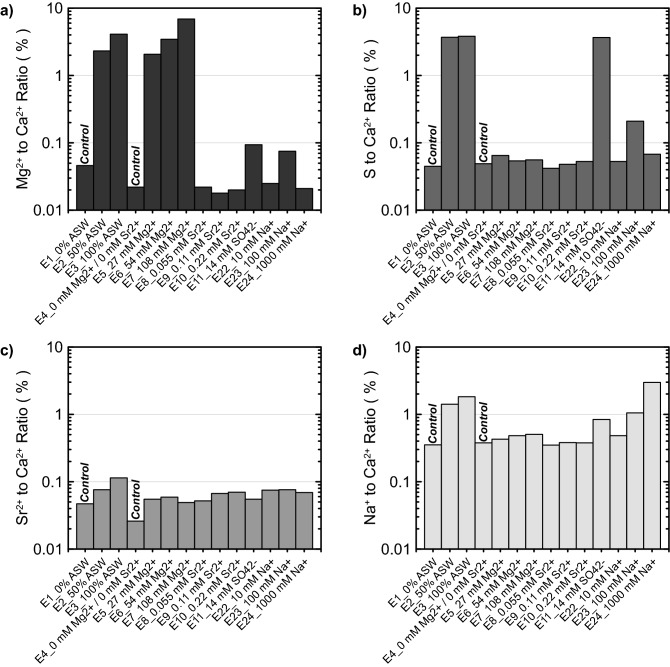
Comparison of (**a**) Mg^2+^ to Ca^2+^ ion ratios, (**b**) S to Ca^2+^ ion ratios, (**c**) Sr^2+^ to Ca^2+^ ion ratios, (**d**) Na^+^ to Ca^2+^ ion ratios obtained from precipitate composition analyses for select experiments.

Precipitate composition analyses were completed for select experiments in order to further characterize the chemical composition of resulting mineral phases. Supplemental Table S2 presents a summary of all results and Fig. 14 presents results obtained from select experiments involving seawater ion additions (experimental series 1, 2, and 4) including comparisons of Mg^2+^-to-Ca^2+^ ion ratios (Fig. 14a), S-to-Ca^2+^ ion ratios (Fig. 14b), Sr^2+^-to-Ca^2+^ ion ratios (Fig. 14c), and Na^+^-to-Ca^2+^ ion ratios (Fig. 14d) within resulting precipitates. Ion concentrations were normalized by Ca^2+^ concentrations in order to examine the abundance of ions as a molar fraction of the total amount of CaCO_3_ dissolved following similar studies^65^. As shown in Fig. 14a, Mg^2+^-to-Ca^2+^ ratios were between 0.02% and 0.05% in control experiments, however, much higher Mg^2+^ abundances were observed in precipitates from the ASW and Mg^2+^ ion varied experiments. Both the 100% ASW and 54 mM Mg^2+^ experiments, which contained similar Mg^2+^ concentrations, had Mg^2+^-to-Ca^2+^ ratios between 2.5 and 4.1%. When Mg^2+^ concentrations were further increased to 108 mM, the highest Mg^2+^-to-Ca^2+^ ratio of 6.9% was observed. These values were consistent with earlier XRD analyses for the ASW and Mg^2+^ varied experiments, which suggested the presence of magnesian calcite through small diffraction peak shifts. Mg^2+^-to-Ca^2+^ ratios for all other experiments were less than 0.09%. Composition analyses also detected varying amounts of sulfur (S) in select precipitates. As shown in Fig. 14b, S-to-Ca^2+^ ratios between 0.04% and 0.05% were present in control experiments with much higher S-to-Ca^2+^ ratios between 3.7% and 3.8% measured in the 14 mM SO_4_^2−^, 50% ASW, and 100% ASW experiments. Although XRD S-Q analyses for these experiments did not detect the presence of sulfate-based minerals (e.g., gypsum), these results suggest that some fraction of precipitates contained sulfur and may have been present at quantities below the threshold of detection (< ≈ 2% by mass) or amorphous in nature. When examining Sr^2+^-to-Ca^2+^ ratios, only small differences between experiments with and without added Sr^2+^ were detected with values near 0.05% in controls and the highest value of 0.11% detected in the 100% ASW experiment (Fig. 14c). Lastly, Na^+^-to-Ca^2+^ ratios were between 0.35% and 0.84% in all experiments without added ASW or Na^+^, however, these ratios progressively increased in both the Na^+^ added and ASW experiments. Na^+^-to-Ca^2+^ ratios were highest in the 1000 mM Na^+^ and 100% ASW experiments which had Na^+^-to-Ca^2+^ ratios of 3.0% and 1.8%, respectively (Fig. 14d). Collectively, these results suggest that Mg^2+^, S, and Na^+^ can be incorporated within precipitates when present in surrounding solutions. This is consistent with observations by Busenberg and Plummer^65^ wherein similar S-to-Ca^2+^ and Na^+^-to-Ca^2+^ ratios were measured in abiotic CaCO_3_ precipitates prepared in ASW. However, Busenberg and Plummer^65^ also found incorporation ratios to depend on the rate of crystal growth, suggesting that changes in ureolytic rates may have also impacted these ratios, if such differences had been considered.

## Conclusions

A study was performed to investigate the effect of marine and brackish conditions and different soil materials on the mineralogy, morphology, and reaction kinetics of ureolytic bio-cementation mediated by augmented *S. pasteurii* bacteria. Thirty-five small-scale batch experiments were completed through five different experiment series and explored the effect of artificial seawater ions, discrete Mg^2+^, Sr^2+^, SO_4_^2−^, and Na^+^ ion additions, and different poorly-graded sands and common soil minerals. During experiments, ureolysis and CaCO_3_ precipitation kinetics were assessed using direct measurements of aqueous urea and Ca^2+^ in time and generated precipitates were characterized using SEM imaging, x-ray diffraction, and precipitate composition analyses. A cell-normalized Michaelis–Menten kinetic model was calibrated to observed urea degradation behaviors using PHREEQC batch reaction models to further characterize the impact of the considered environmental conditions on ureolytic activity, compare activity-based cell density estimations to those measured directly via optical density measurements, and explored expected relationships between aqueous urea and Ca^2+^ concentrations during precipitation. From the results of this study, the following conclusions can be made:

- Although differences in precipitate morphology and mineralogy were observed with changes in the examined geochemical conditions, in all experiments calcite was the predominant CaCO_3_ mineral polymorph with relative percentages exceeding 80% by mass. Factors found to decrease calcite and increase vaterite and aragonite relative percentages included increases in artificial seawater and supplied Mg^2+^ concentrations.
- Artificial seawater experiments exhibited significant reductions in ureolysis and precipitation rates and exhibited distinct crystal morphologies, despite having similar mineralogical compositions as control experiments. Additional experiments containing minimal added Ca^2+^ (< 10 mM) further confirmed that the reaction inhibition observed in the 100% ASW experiment could be primarily attributed to the effect of Mg^2+^ on urea hydrolysis activity with some smaller fraction likely related to the higher ionic strength of seawater. Precipitate composition analyses further indicated that precipitates formed in artificial seawater solutions contained Mg^2+^, Sr^2+^, S, and Na^+^ substitutions. In experiments containing added SO_4_^2−^ and Sr^2+^, however, no significant differences in reaction kinetics or precipitate mineralogy or morphology were observed for the concentrations considered (Sr^2+^  < 0.22 mM, SO_4_^2−^  < 14 mM).
- Aqueous urea and Ca^2+^ concentrations measured in time during all experiments were well approximated by modeled trends which assumed CaCO_3_ precipitation to be an equilibrium reaction. When geochemical conditions similar to those considered in this study are present, CaCO_3_ precipitation during MICP may be assumed to be an equilibrium reaction and eliminate the need for other computationally intensive kinetic expressions for CaCO_3_ precipitation to be incorporated in geochemical models.
- Quantitative comparisons of activity-based cell density estimates between all experiments and their respective controls suggested that the most significant inhibition of ureolysis occurred in experiments containing 100% ASW, 108 mM Mg^2+^, and Fraser River Sand with ureolytic activities exceeding rates observed in the control experiments in only the montmorillonite and mica containing specimens. Moving forward, characterization of Mg^2+^ concentrations and other exchangeable ions in groundwater and field soils may be critical towards assessing potential inhibition of bio-cementation.
- Comparisons of activity-based cell density estimates and direct measurements of augmented cell densities suggested that significant discrepancies existed between direct cell density measurements and observed bulk ureolytic activities for all experiments including controls. Moving forward, activity-based characterizations such as those afforded by direct urea concentration measurements will likely provide superior insights for field applications when compared to direct measurements of augmented cell densities such as those from OD_600_, total direct counts, or total protein measurements.

While the obtained results provide important insights regarding the influence of seawater ions and varying soil materials on the reaction kinetics and physicochemical properties of bio-cemented soils, further research is needed to more fully understand the effect of a variety of other important practical conditions including changes in treatment solution composition and biological treatment strategies.

## Supplementary Information

Supplementary Information.

## Data Availability

All data generated during the study are available from the corresponding author upon reasonable request. All data presented in the figures of this paper as well as experimental measurements be available through the NSF DesignSafe-CI Data Depot repository (https://www.designsafe-ci.org/data/browser/public/) under project number PRJ-3190.
