# Supplementary material for: Mineralogy, morphology, and reaction kinetics of ureolytic bio-cementation in the presence of seawater ions and varying soil materials
Source: Sci Rep. 2022 Oct 12;12:17100. doi: 10.1038/s41598-022-21268-3 (PMC9556692; doi:10.1038/s41598-022-21268-3)
Supplement: Supplementary file 1 — Supplementary Information. [file 41598_2022_21268_MOESM1_ESM.pdf]

Supplementary Information

Title: Mineralogy, Morphology, and Reaction Kinetics of Ureolytic Bio-cementation in the Presence of Seawater Ions and Varying Soil Materials

Authors: Robert J. Burdalski[1], Bruna G.O. Ribeiro[2], Michael G. Gomez[3*], Drew Gorman-Lewis[4]

[1]M.S. Student, Department of Civil and Environmental Engineering, University of Washington, Seattle, WA 98195, e-mail: bob.burdalski@gmail.com

[2] Ph.D. Student, Department of Civil and Environmental Engineering, University of Washington, Seattle, WA 98195, e-mail: brunagr@uw.edu, ORCID: 0000-0003-3501-9089

[3*]Assistant Professor, Department of Civil and Environmental Engineering, University of Washington, Seattle, WA, 98195, email: mggomez@uw.edu, ORCID: 0000-0002-4464-544 ([*]Corresponding Author)

[4]Associate Professor; Department of Earth and Space Science, University of Washington, Seattle, WA, 98195, email: dgormanl@uw.edu, ORCID: 0000-0002-9687-7039

Supplemental Table S1. Summary of Soil Exchangeable Cations and Cation Exchange Capacities for Tested Soil Materials

		Exchangeable Ions[1,2]																
	CEC (meq/100 g)	Al (µg/g)	As (µg/g)	B (µg/g)	Ba (µg/g)	Ca (µg/g)	Cu (µg/g)	Fe (µg/g)	K (µg/g)	Mg (µg/g)	Mn (µg/g)	Mo (µg/g)	Na (µg/g)	Ni (µg/g)	S (µg/g)	Zn (µg/g)	Si (µg/g)	Ag (µg/g)
Ottawa Sand	0.17	-	-	-	0.3	43.6	-	TR	TR	TR	0.2	-	8.3	-	7.3	-	TR	-
Fraser River Sand	0.87	35.6	TR	0.4	5.3	90.5	1.2	71.3	112.3	179.6	4.8	TR	159.5	4.5	235.2	2.1	34.8	-
Concrete Sand	2.58	TR	-	0.6	21.4	277.7	-	-	18.9	191.5	TR	0.5	23.9	0.2	3.6	-	9.9	TR
Covelo Sand	1.64	0.7	-	TR	9.4	555	-	-	18.7	59.6	1.3	TR	5.7	TR	17	2.3	4.8	TR
Delta Sand	4.32	TR	-	0.3	13.5	237	-	-	84	238.8	1.5	0.3	180.1	0.4	81.8	TR	9.6	-
Monterey Sand	0.08	11.9	TR	TR	0.2	16	-	3.8	36.3	31.3	0.4	-	139.1	TR	12.6	-	6.3	-
Feldspar	0.39	4.4	-	TR	0.6	42	-	3	TR	4.1	0.7	-	5.4	-	4.5	-	3.3	-
Olivine	0.29	-	-	-	TR	16	-	1.9	TR	92.2	0.6	-	-	1	TR	-	33.3	-
Mica	9.24	15.9	-	-	0.8	3	-	10.7	33.5	4.6	6.1	-	8.7	TR	3.9	-	13.5	-
Kaolinite	3.42	13.6	-	TR	2.3	3630	TR	1.3	133.4	110.9	1.6	TR	35.3	0.3	3342	-	50.6	0.5
Montmorillonite	50.2	1.9	1.2	0.4	3.8	2615	TR	-	382.7	602.7	0.6	2	-	0.7	765.7	-	197.6	TR

[1] "-" denotes non-detectable concentrations

[2] "TR" denotes trace concentrations detected (< 0.2 µg/g)

Supplemental Table S2. Summary of Precipitate Composition Analysis Results

Plate Experiments			Composition of Dissolution Solution (15 mM HCl) After Equilibration[1,2]																			
Experiment Name	Experimental Series	Soil Material	Ag (µM)	Al (µM)	As (µM)	B (µM)	Ba (µM)	Ca (µM)	Cu (µM)	Fe (µM)	K (µM)	Mg (µM)	Mn (µM)	Na (µM)	Ni (µM)	P (µM)	Pb (µM)	S (µM)	Se (µM)	Si (µM)	Sr (µM)	Zn (µM)
E1_0% ASW	(1) ASW	Ottawa F-65 Sand	1.01	0.68	-	-	0.03	5367.03	TR	0.23	53.07	2.49	TR	18.95	-	3.31	-	2.41	-	4.19	2.51	1.68
E2_50% ASW	(1) ASW	Ottawa F-65 Sand	1.01	0.92	-	0.86	0.03	5072.61	0.02	0.25	27.62	117.63	0.03	71.51	-	3.49	-	187.59	TR	3.66	3.88	0.57
E3_100% ASW	(1) ASW	Ottawa F-65 Sand	0.96	0.59	-	1.42	0.03	4890.46	0.04	0.35	57.83	200.95	0.03	89.26	-	3.22	-	186.68	-	4.90	5.59	1.03
E4_0 mM Mg2+ / 0 mM Sr2+	(2) ASW Ions	Ottawa F-65 Sand	0.96	1.25	-	-	TR	4845.55	0.03	0.26	51.31	1.06	TR	18.29	-	2.55	-	2.37	-	4.84	1.26	0.43
E5_27 mM Mg2+	(2) ASW Ions	Ottawa F-65 Sand	0.88	1.90	-	-	0.09	4373.97	0.05	0.86	68.37	90.35	0.03	18.76	-	2.58	TR	2.83	TR	5.39	2.40	0.61
E6_54 mM Mg2+	(2) ASW Ions	Ottawa F-65 Sand	0.85	1.22	0.02	0.67	0.07	4037.13	0.05	0.64	84.68	139.77	0.05	19.41	TR	1.85	TR	2.18	TR	5.13	2.40	0.39
E7_108 mM Mg2+	(2) ASW Ions	Ottawa F-65 Sand	0.90	0.58	TR	0.23	0.06	4421.38	0.08	0.47	87.78	304.92	0.03	22.28	-	2.51	TR	2.49	-	5.17	2.17	0.47
E8_0.055 mM Sr2+	(2) ASW Ions	Ottawa F-65 Sand	0.92	1.25	-	-	0.04	4803.13	0.06	0.25	94.71	1.06	0.02	16.75	-	2.53	TR	2.03	TR	5.50	2.51	0.51
E9_0.11 mM Sr2+	(2) ASW Ions	Ottawa F-65 Sand	0.88	1.33	-	-	0.05	4458.81	0.04	0.26	97.29	0.82	0.02	16.99	-	2.53	TR	2.15	-	4.41	2.97	0.40
E10_0.22 mM Sr2+	(2) ASW Ions	Ottawa F-65 Sand	0.89	1.41	-	-	0.06	4386.45	0.05	0.30	96.45	0.86	0.03	16.56	-	2.37	TR	2.34	-	4.27	3.08	0.52
E11_14 mM SO42-	(2) ASW Ions	Ottawa F-65 Sand	0.96	3.95	-	0.16	0.39	4967.81	0.07	2.13	100.06	4.65	0.15	41.81	-	2.51	TR	182.22	-	10.82	2.74	1.24
E22_10 mM Na+	(4) Sodium Variations	Ottawa F-65 Sand	0.91	1.41	-	-	0.12	4690.85	0.06	0.97	103.97	1.17	0.04	22.57	TR	3.66	TR	2.47	-	4.84	3.54	0.91
E23_100 mM Na+	(4) Sodium Variations	Ottawa F-65 Sand	0.97	1.30	-	-	0.58	4962.82	0.02	0.85	96.09	3.74	0.13	52.11	-	3.69	-	10.44	0.03	9.94	3.77	1.45
E24_1000 mM Na+	(4) Sodium Variations	Ottawa F-65 Sand	0.86	1.51	-	-	0.09	4314.09	0.03	0.49	96.73	0.88	0.02	128.27	-	3.09	TR	2.94	TR	4.05	2.97	0.45
E25_Ottawa Sand	(5) Soil Variations	Ottawa F-65 Sand	0.90	0.77	-	-	0.10	4596.04	-	0.19	129.57	0.71	TR	19.37	-	4.12	TR	2.50	-	3.57	3.31	0.23
E26_Fraser River Sand	(5) Soil Variations	Fraser River Sand	0.90	12.74	-	3.58	0.13	4471.28	0.45	8.07	158.75	20.33	0.65	31.66	0.44	10.64	TR	21.16	-	87.09	3.65	0.86
E27_Concrete Sand	(5) Soil Variations	Concrete Sand	1.03	0.72	-	2.74	0.39	5404.46	TR	0.15	148.29	14.83	1.55	22.82	0.05	2.81	-	2.06	-	26.31	3.77	0.24
E28_Covelo Sand	(5) Soil Variations	Covelo Sand	1.04	0.03	-	3.64	0.33	5459.35	TR	0.21	111.87	13.45	1.03	22.75	0.09	2.49	-	10.31	-	25.81	4.79	0.74
E29_Delta Sand	(5) Soil Variations	Delta Sand	0.95	0.61	-	3.99	0.31	4828.09	-	0.72	112.41	18.50	3.75	24.72	0.11	1.73	-	21.23	-	71.82	5.02	0.26
E30_Monterey Sand	(5) Soil Variations	Monterey Sand	0.87	0.73	-	0.68	0.07	4156.89	0.08	0.28	169.16	5.25	0.07	24.25	-	1.75	-	1.86	-	6.27	4.79	0.72
E31_Feldspar	(5) Soil Variations	Feldspar	0.81	1.47	TR	-	0.07	3805.08	0.03	0.20	91.33	0.68	0.07	17.11	-	2.47	TR	1.69	-	2.29	2.62	0.94
E32_Olivine	(5) Soil Variations	Olivine	0.85	0.14	-	0.72	0.08	4329.06	TR	0.74	139.90	46.12	0.14	17.81	0.33	1.58	-	2.18	-	32.33	4.11	0.25
E33_Mica	(5) Soil Variations	Mica	0.95	0.30	-	0.06	0.06	5152.45	-	0.07	104.30	0.83	0.17	27.68	-	8.07	-	2.13	-	4.98	3.65	0.34
E34_Kaolinite	(5) Soil Variations	Kaolinite	0.99	-	-	5.01	0.12	5352.06	TR	0.05	181.67	2.31	0.03	27.93	-	3.69	-	8.00	-	60.64	3.54	0.08
E35_Montmorillonite	(5) Soil Variations	Montmorillonite	0.92	-	-	2.69	0.75	4863.02	-	TR	108.83	36.06	1.29	54.33	TR	7.19	-	26.00	TR	134.98	3.20	TR

[1] "-" denotes non-detectable concentrations

[2] "TR" denotes trace concentrations (< 0.02 µM)

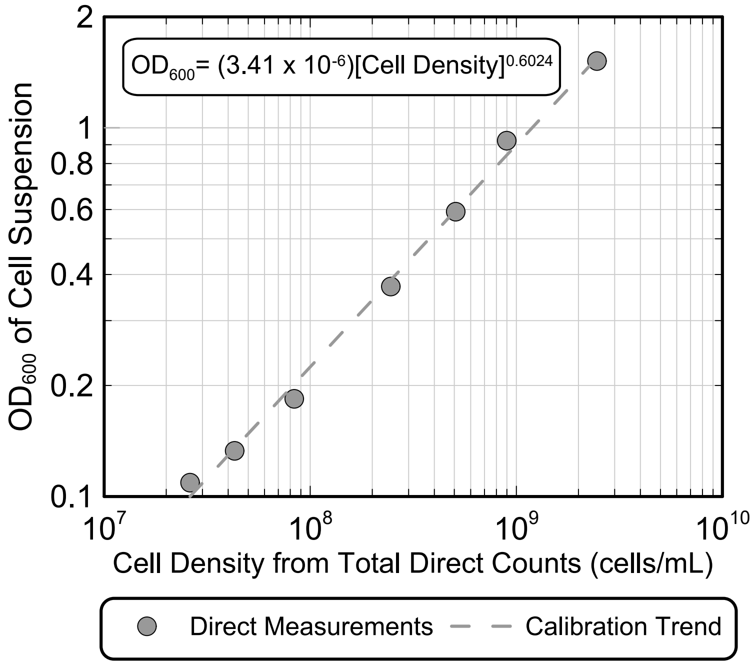

Supplemental Figure S1. Lab-specific correlation between optical density measurements at 600 nm (OD_{600}) and total direct cell counts using epifluorescence microscopy for similar cell suspensions of *S. pasteurii* prepared at varying cell densities.

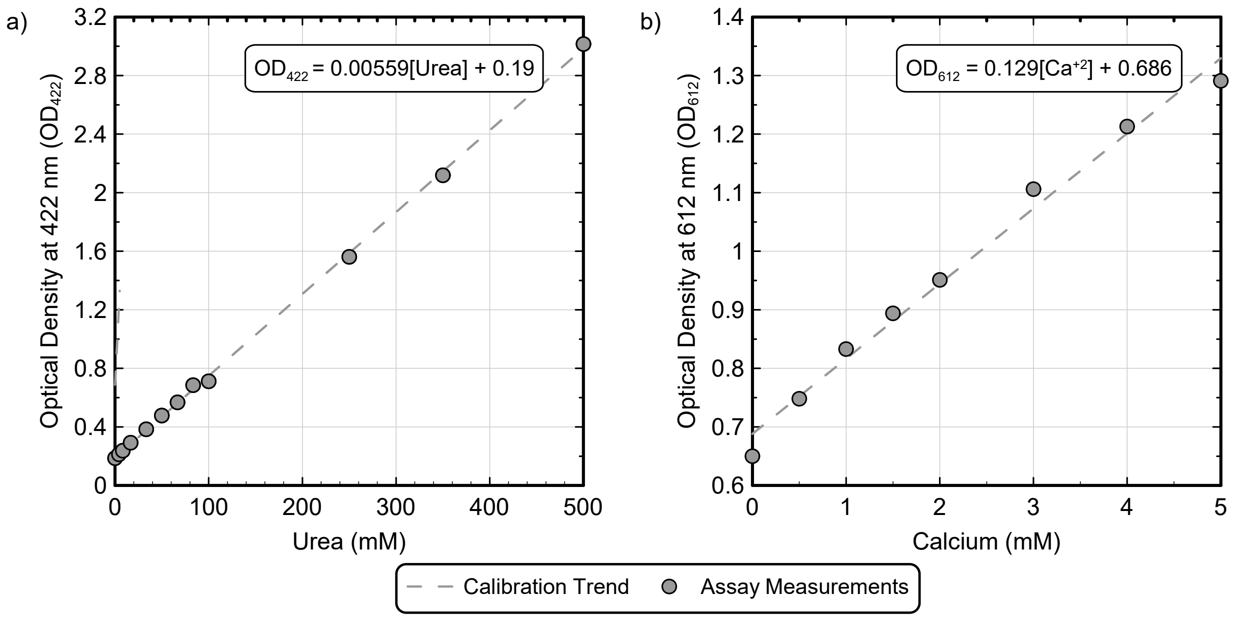

Supplemental Figure S2. Example calibration relationships for **(a)** urea and **(b)** Ca^{2+} concentration assays which were used to determine unknown concentrations of urea and Ca^{2+} within samples. All urea and Ca^{2+} assay measurements were completed using a spectrophotometer at wavelengths of 422 nm and 612 nm, respectively. New calibration curves were generated prior to completing all aqueous measurements reported in this study.

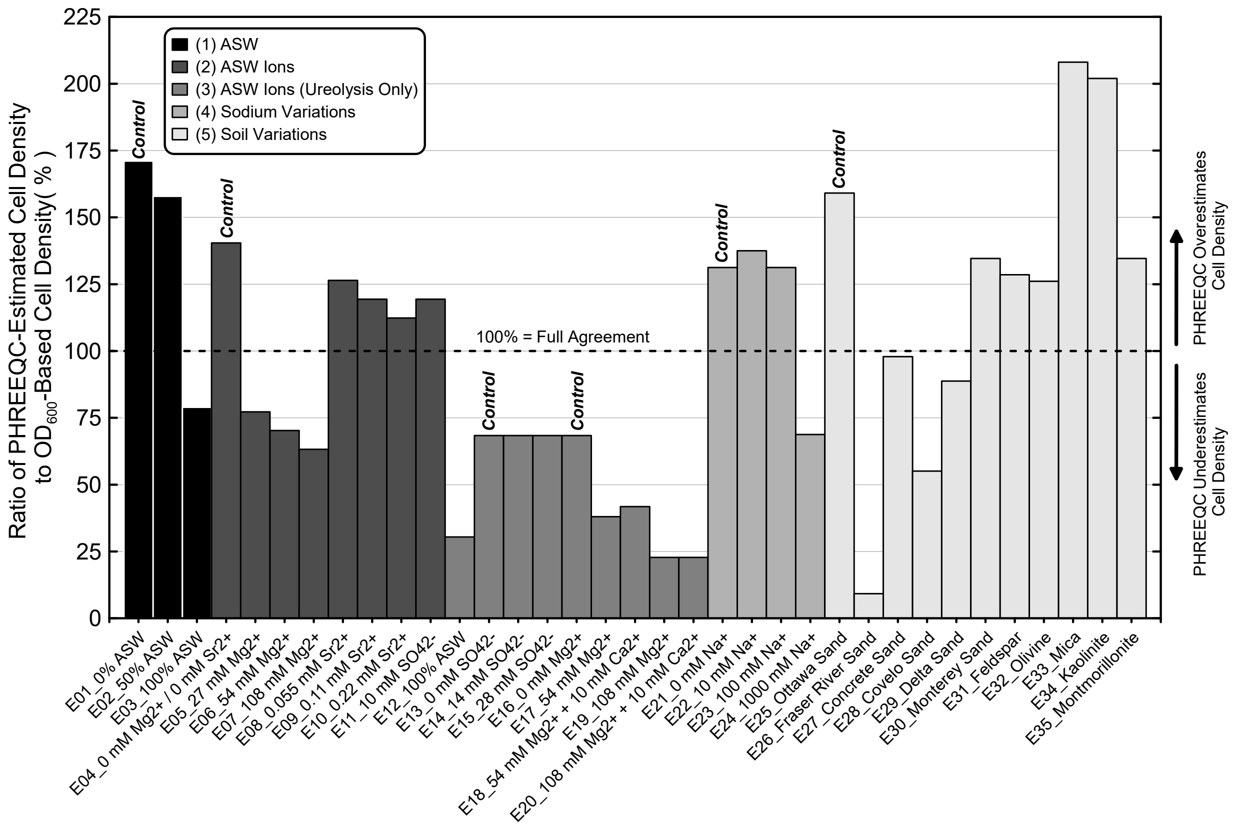

Supplemental Figure S3. Ratio of PHREEQC-estimated activity-based *S. pasteurii* cell densities to direct OD_{600}-based measurements of *S. pasteurii* cell densities for all experiments.

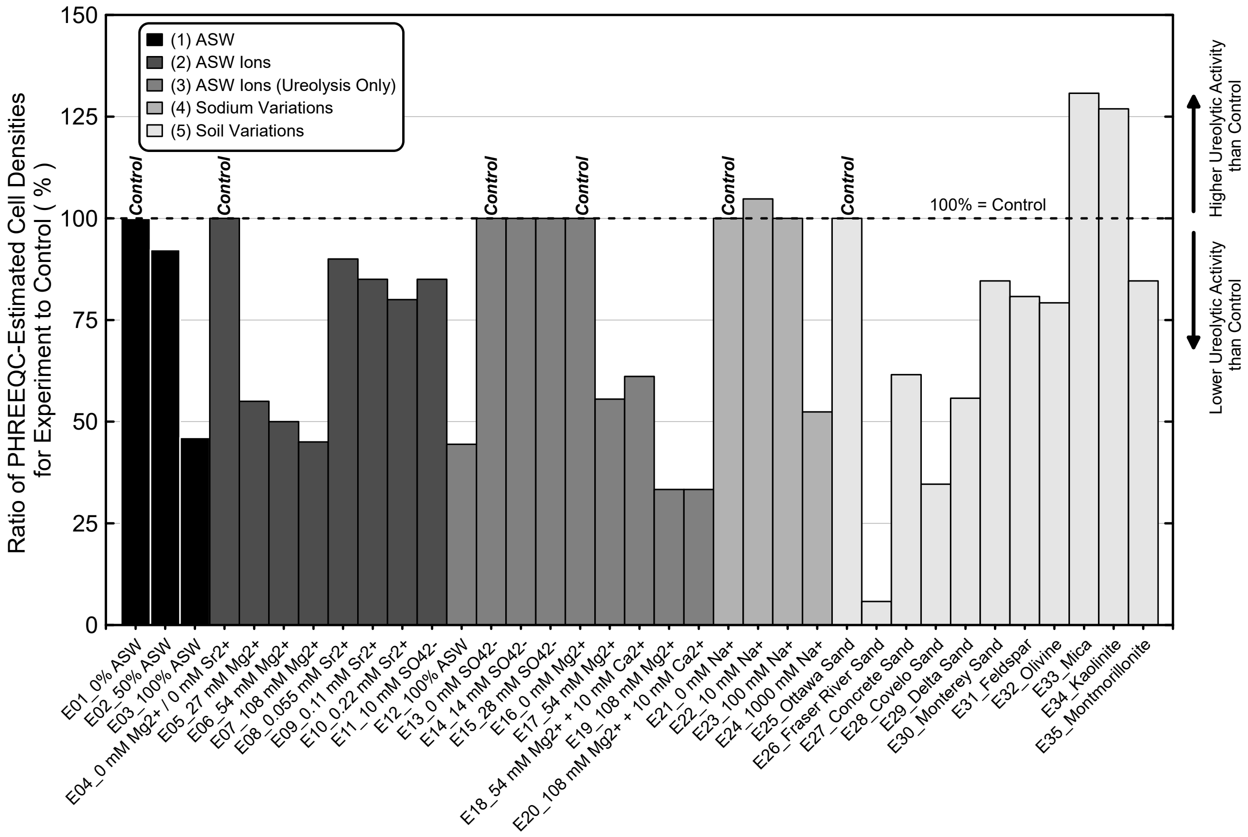

Supplemental Figure S4. Ratio of PHREEQC-estimated activity-based *S. pasteurii* cell densities for all experiments to PHREEQC-estimated activity-based *S. pasteurii* cell densities for respective control experiments (E01, E04, E16, E21, E25) from the same experimental series.
